# Efficacy and Safety of Orforglipron in Obese Adults With or Without Diabetes: A Systematic Review and Meta‐Analysis

**DOI:** 10.1002/edm2.70134

**Published:** 2025-11-26

**Authors:** Ravi Kumar Pandey, Mahnoor Jan, Aqsa Mohammad, Khawaja Arham Jawaid, Mawra Naveed, Muhammad Ali Abid, Abdullah Bin Amir, Shafique Ahmed, Muhammad Safiullah, Muhammad Imaz Bhatti, Sana Iftikhar, Muhammad Nabeel Saddique, Widyan Alfalh, Rihab Mohammed bin Omar, Husam Abu Dawood

**Affiliations:** ^1^ Nepalgunj Medical College Nepalgunj Lumbini Province Nepal; ^2^ Shaikh Khalifa Bin Zayed Al‐Nahyan Medical and Dental College Lahore Pakistan; ^3^ Rawalpindi Medical University Rawalpindi Pakistan; ^4^ Sharif Medical City Hospital Lahore Pakistan; ^5^ King Edward Medical University Lahore Pakistan; ^6^ International University of Kyrgyzstan Bishkek Kyrgyzstan; ^7^ Dar Alhekma Hospital Benghazi Libya; ^8^ University of Tripoli Tripoli Libya; ^9^ Palestine Polytechnic University Hebron Palestine

**Keywords:** GLP‐1 receptor agonist, glycemic control, meta‐analysis, obesity, orforglipron, type 2 diabetes mellitus

## Abstract

**Background:**

Obesity represents a major global health challenge, contributing substantially to cardiovascular disease and metabolic complications. Orforglipron, a novel oral non‐peptide GLP‐1 receptor agonist, has emerged as a promising therapeutic option for weight management and glycemic control. This meta‐analysis evaluated the efficacy and safety of orforglipron in obese patients with or without type 2 diabetes mellitus (T2DM).

**Methods:**

A comprehensive literature search was conducted across PubMed, Embase, Cochrane Library and ClinicalTrials.gov from inception to October 2025 to identify randomised controlled trials (RCTs) evaluating orforglipron in obese patients. Mean differences (MDs) and risk ratios (RRs) with 95% confidence intervals (CIs) were calculated using random‐effects models.

**Results:**

Five RCTs comprising 4410 participants were included. Orforglipron demonstrated dose‐dependent reductions in body weight from 2.48% at 3 mg to 9.8% at 45 mg, BMI from 0.89 to 3.62 kg/m^2^, waist circumference from 1.57 to 6.9 cm, and HbA1c from 0.76% to 1.04% compared with placebo. Favourable lipid changes included reductions in total cholesterol (MD: −4.51% [95% CI: −6.91 to −2.11]), LDL‐C (MD: −5.34% [95% CI: −7.10 to −3.58]), and triglycerides (MD: −10.07% [95% CI: −12.33 to −7.80]), with increased HDL‐C (MD: 2.94% [95% CI: 1.38 to 4.49]). However, gastrointestinal adverse events were significantly more frequent at doses of 12 mg or higher and treatment discontinuation rates were highest at 24 mg (RR:4.61 [95% CI:1.6 to 13.33]) and 36 mg (RR:3.68 [95% CI:2.48 to 5.44]) doses. Serious adverse events and mortality rates were comparable to those with placebo.

**Conclusion:**

Orforglipron significantly improved glycemic and lipid parameters in patients with obesity, demonstrating dose‐dependent efficacy with maximal benefits at higher doses. While gastrointestinal tolerability remains a clinically important limitation requiring mitigation strategies, orforglipron represents a promising oral therapeutic option for comprehensive obesity and metabolic management.

## Graphical abstract

This systematic review and meta‐analysis of 5 randomised controlled trials (4410 participants) demonstrates that orforglipron produces dose‐dependent reductions in HbA1c, BMI, body weight, and waist circumference in obese adults. Higher doses (24–45 mg) show greater efficacy, supporting orforglipron's promising role in managing obesity and improving metabolic parameters.

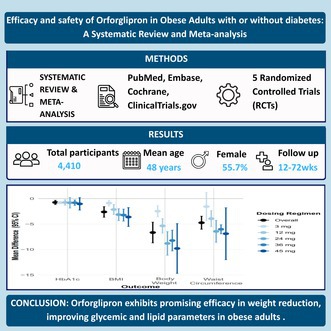

## 1 Introduction

Obesity is defined as abnormal or excessive fat accumulation that poses a significant health risk, primarily characterised by a body mass index (BMI) of 30 kg/m^2^ or higher [1]. This progressive multifactorial disease has reached epidemic proportions worldwide, affecting both developed and developing nations. Current epidemiological evidence suggests that globally there are over 600 million adults classified as obese, representing a nearly three‐fold increase since 1975 [2]. According to the World Health Organization (WHO) estimates, in 2022 approximately 2.5 billion adults (43% of the global adult population) aged 18 years or older were overweight, with 890 million individuals (16%) meeting the criteria for obesity [3].

Obesity is linked to numerous health consequences such as type 2 diabetes mellitus (T2DM), cardiovascular diseases (CVD), hypertension, dyslipidemias, certain cancers, fatty liver disease, osteoarthritis, and obstructive sleep apnea [4, 5]. These comorbidities substantially compromise quality of life (QoL), increase disability, and result in higher all‐cause mortality, marking obesity as a major public health concern that contributes to a greater disease burden and economic costs [4]. The economic impact of obesity extends beyond direct healthcare expenditures such as medical care, hospitalizations, medications as well as indirect costs like less productivity, absenteeism, and premature mortality. Obesity is estimated to account for approximately 2%–3% of global GDP in healthcare and productivity losses, with forecasts suggesting this figure could exceed 3% by 2060 if current trends continue [6, 7].

The management of obesity requires a multifaceted approach that includes lifestyle modification, pharmacotherapy, and bariatric surgery [8, 9]. Bariatric surgery is effective in achieving substantial and lasting weight loss (up to 25%); however, its invasive nature, high cost, surgical risks, and restrictive eligibility criteria significantly limit accessibility. Additional barriers include potential complications, long‐term nutritional deficiencies, and the requirement for lifelong medical surveillance [9]. Pharmacotherapy offers an intermediate strategy with GLP‐1 receptor agonists (mainly injectables), orlistat, and combination therapies such as naltrexone‐bupropion and phentermine‐topiramate [8, 10]. GLP‐1 receptor agonists, including liraglutide, semaglutide, and tirzepatide (dual GIP/GLP agonists), have revolutionised the field of obesity pharmacotherapy by facilitating substantial weight loss, achieving an average reduction of 9% with liraglutide, up to 15% with semaglutide, and 18% with tirzepatide [11, 12]. In the context of emerging dual GIP/GLP 1 receptor agonists, the development of orforglipron represents an important advancement. As a potent oral GLP 1 receptor agonist, orforglipron offers the potential to enhance accessibility, simplify administration, and improve long‐term adherence in the management of obesity [13, 14]. Nevertheless, its use is constrained by the subcutaneous administration route, high costs, side effects, and adherence difficulties [11, 15].

The emergence of oral semaglutide provided an alternative to injectable GLP‐1 RAs. Yet, its adoption is restricted by a complicated dosing protocol that necessitates administration while fasting with water, as well as the avoidance of food or medications for a minimum of 30 min afterward. Such requirements reduce convenience and compromise adherence in routine practice [11, 16]. Oral semaglutide co‐formulation with sodium N‐(8‐[2‐hydroxybenzoyl] amino) caprylate (SNAC), a pharmaceutical absorption enhancer facilitates peptide absorption through three distinct mechanisms: (1) neutralisation of the local gastric pH microenvironment, thereby protecting semaglutide from enzymatic degradation; (2) reduction of semaglutide oligomerization; and (3) transient, reversible enhancement of gastric epithelial membrane permeability to enable transcellular drug absorption. Despite the transient nature of SNAC‐mediated permeability changes, clinical studies have demonstrated that oral semaglutide increases levothyroxine systemic exposure by 33%. This clinically significant drug interaction necessitates careful monitoring of thyroid function parameters in patients receiving concurrent thyroid hormone replacement therapy [17].

Orforglipron (LY3502970), first‐in‐class oral, non‐peptide partial agonist of the GLP‐1 receptor that favors activation of G protein over β‐arrestin recruitment. Unlike oral semaglutide, orforglipron allows for once‐daily oral dosage without fasting or water intake restrictions, potentially enhancing both adherence and accessibility [18]. Orforglipron exerts GLP‐1 mediated effects by suppressing appetite, delaying gastric emptying, and improving glycemic control [18]. Phase 2 Randomised Clinical Trials(RCTs) have reported significant weight reduction of up to 14.7% at 36 weeks, alongside improvements in cardiometabolic markers in patients with obesity and type 2 diabetes, demonstrating an efficacy and safety profile comparable to injectable liraglutide and superior to oral semaglutide at standard doses [18, 19].

Given its promising weight loss efficacy and favourable cardiometabolic profile, orforglipron may offer a transformative therapeutic option for obesity management. However, the evidence is currently scattered among individual RCTs. Therefore, this systematic review and meta‐analysis aims to evaluate the safety and efficacy of orforglipron in obese patients with and without diabetes mellitus.

## 2 Methods

This study‐level meta‐analysis was carried out in compliance with the Cochrane Handbook for Systematic Reviews of Interventions guidelines and reported according to the PRISMA checklist (Preferred Reporting Items for Systematic Reviews and Meta‐Analyses) [20, 21]. The protocol for this study was registered with PROSPERO under registration number CRD420251151562. The PRISMA checklist is given in Table S1.

### 2.1 Literature Search and Search Strategy

A comprehensive systematic literature search was conducted on PubMed, Embase, Cochrane, and ClinicalTrials.gov from inception to October 2025, using appropriate keywords and their Medical Subject Headings (MeSH) terms: “orforglipron” and “LY3502970”. The detailed search strategy, incorporating Medical Subject Headings (MeSH) terms, and keywords is outlined in Table S2.

### 2.2 Eligibility Criteria

Studies were included if they met the following criteria: (1) were randomised controlled trials (RCTs); (2) evaluated the efficacy or safety of orforglipron in obese patients (≥ 18 years) with or without diabetes; (3) involved administration of orforglipron at doses of 3, 12, 24, 36 or 45 mg; (4) compared orforglipron with placebo; and (5) reported at least one relevant outcome of interest.

Studies were excluded if they (1) were not RCTs; (2) did not provide sufficient data for analysis; (3) were animal‐based; or (4) were not published in English.

### 2.3 Study Selection Process and Data Extraction

All records were imported into Rayyan where duplicates were removed. Two authors (R.K.P. and M.J.) independently screened the articles based on title, and abstracts, excluding irrelevant studies. Full texts of the remaining articles were subsequently reviewed based on predefined inclusion criteria. Any disagreements or conflicts were resolved through discussion with a third independent reviewer (MS).

Three independent reviewers (K.A.J., A.B.A., and S.A.) extracted data from the included studies, focusing on study characteristics (authorship, publication year, study design, trial type, and follow‐up duration), population details (age and gender), interventions, comparators, and efficacy and safety outcomes. The data were entered into a standardised Excel sheet. Any discrepancies were resolved by a fourth independent reviewer (M.S.). Data presented in graphical formats were digitised using WebPlotDigitizer, a web‐based tool [22].

### 2.4 Quality Assessment

The revised Cochrane Risk of Bias tool for randomised trials (RoB 2.0) [23], outlined in the Cochrane Handbook was used to assess risk of bias among included studies. Each study was independently evaluated across five domains, including: (1) the randomization process, (2) deviations from intended interventions, (3) missing outcome data, (4) outcome measurement, and (5) selection of reported results. Studies were categorised as having a low risk of bias, high risk of bias, or some concerns. A visual summary of the risk of bias assessment was generated using the Robvis tool.

### 2.5 Outcomes and Statistical Analysis

The efficacy outcomes included change in HbA1c %, fasting serum glucose mg/dl, percentage body weight change, body weight change in kg, change in BMI (kg/m^2^), waist circumference in cm, % change in serum total cholesterol, % change in LDL total cholesterol, % change in HDL cholesterol, and % change in triglycerides. Safety outcomes were any TEAE (treatment‐emergent adverse events), serious adverse events, adverse events resulting in discontinuation, and death.

Statistical analyses were performed using Review Manager (RevMan, version 5.4; The Cochrane Collaboration, Copenhagen, Denmark). When studies reported medians with interquartile ranges (IQRs) or 95% CI, these values were transformed to means and standard deviations (SDs) utilizing the Meta‐Converter web‐based application [24]. Pooled outcome estimates were expressed as mean differences (MD) and risk ratios (RR), along with 95% CI. Quantitative synthesis was performed using random‐effects modeling with the inverse variance weighting method for continuous and Mantel–Haenszel method (M‐H) for dichotomous variables. Higgins *I* ^2^ statistics were used to evaluate study heterogeneity. *I* ^2^ values < 50% indicate low heterogeneity, *I* ^2^ values of 50%–75% indicate moderate heterogeneity, and *I* ^2^ values > 75% indicate significant heterogeneity. A *p*‐value of < 0.05 represented statistical significance.

For overall pooled effect, we combined multiple arms given in one study into a single group to prevent overestimation of pooled estimates due to double counting of placebo (unit of analysis error). Subgroup analyses were performed to explore the effects of 3, 12, 24, 36 and 45 mg orforglipron on clinical outcomes separately. Funnel plots were not used for assessing publication bias, as the recommended minimum of 10 studies per outcome was not met.

## 3 Results

### 3.1 Study Selection

**FIGURE 1:**
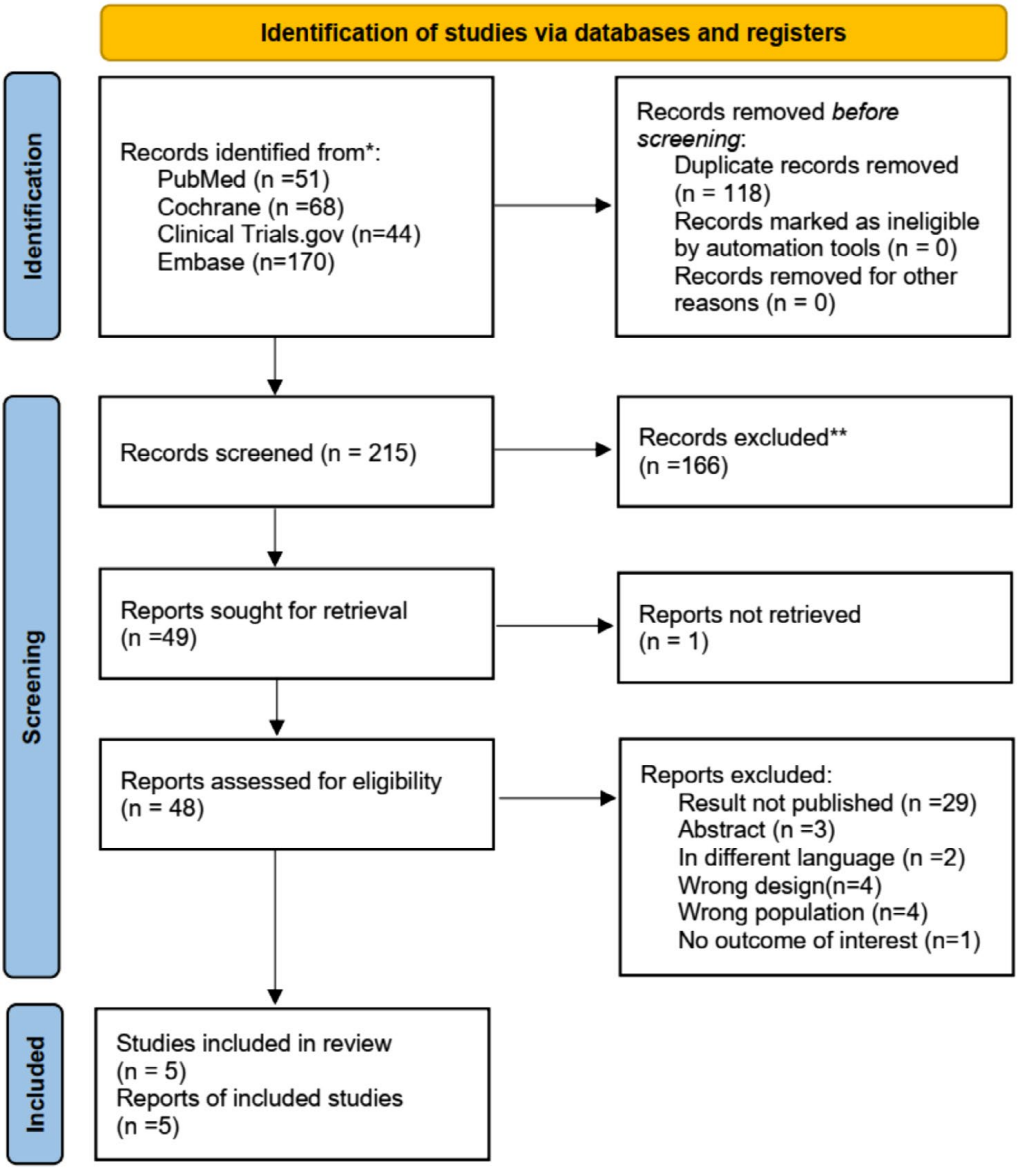
PRISMA flowchart.

A total of 333 studies were identified, out of which 118 were removed as duplicates. The remaining 215 studies were screened based on their titles and abstracts, resulting in the exclusion of 166 studies. The remaining 49 full‐text articles were assessed for eligibility, and five [19, 25, 26, 27, 28] met the inclusion criteria and were included in the analysis. The detailed study selection process is depicted in the PRISMA flow diagram (Figure 1).

### 3.2 Baseline Characteristics and Patient Demographics

Five randomised controlled trials (RCTs) comprising 4410 patients were included. The mean age across the studies was 48 ± 12.6 years, with 61.6% of the participants female. The mean Body Mass Index (BMI) across studies was 36.3 ± 6.8 kg/m^2^, with a mean body weight of 100.8 ± 22.4 kg and a mean waist circumference of 112.1 ± 15.9 cm. The duration of follow‐up ranged from 12 to 72 weeks. Characteristics of the included studies are summarised in Table 1 and baseline values are given in Table S3.

**TABLE 1:** Demographical characteristics of included studies.

Study ID	Country	Arms Evaluated	Sample size (*n*)	White *n* (%)	Black *n* (%)	Asian *n* (%)	Age, years (mean ± SD)	Female *n* (%)	Duration
Frias et al. 2023	United States, Hungary, Poland, Slovakia	*3 mg*	51	47 (92)	2 (4)	1 (2)	59 **±** 9.43	25 (49%)	28 weeks
		*12 mg*	56	49 (88)	5 (9)	1 (2)	57.4 **±** 9.23	20 (36%)	
		*24 mg*	47	43 (91)	2 (4)	1 (2)	60.50 **±** 9.11	17 (36%)	
		*36 mg*	61	58 (95)	0	1 (2)	59.7 **±** 9.2	25 (41%)	
		*45 mg*	63	57 (90)	5 (8)	0	58.5 **±** 9.4	23 (37%)	
		*Placebo*	55	50 (91)	4 (7)	0	58.3 **±** 9.52	27 (49%)	
Rosenstock et al. 2025	India, Mexico, Japan, United States	*3 mg*	143	37 (25.9)	8 (5.6)	63 (44.1)	53.3 ± 11.3	63 (44.1)	42 weeks
		*12 mg*	137	30 (21.9)	9 (6.6)	59 (43.1)	54.1 ± 11.8	71 (51.8)	
		*24 mg*	141	40 (28.4)	4 (2.8)	62 (44.0)	52.8 ± 11.8	72 (51.1)	
		*Placebo*	138	38 (27.5)	3 (2.2)	61 (44.2)	53.3 ± 12.5	63 (45.7)	
Pratt et al. 2023	United States, Germany	*9 mg*	9	NR	NR	NR	57.7 ± 6.4	5 (55.6)	12 weeks
		*15 mg*	10	NR	NR	NR	59.6 ± 4.6	3 (30.0)	
		*21 mg*	14	NR	NR	NR	55.3 ± 8.0	4 (28.6)	
		*27 mg*	9	NR	NR	NR	58.8 ± 4.6	2 (22.2)	
		*45 mg*	9	NR	NR	NR	62.8 ± 4.4		
		*Placebo*	17	NR	NR	NR	56 ± 6.0	7 (41.2)	
Wharton et al. 2023	United States, China, Japan, India, Mexico	*12 mg*	50	47 (94)	3 (6)	0	49.8 ± 10.5	31 (62)	40 weeks
		*24 mg*	53	46 (87)	6 (11)	0	57 ± 9.1	30 (57)	
		*36 mg*	58	50 (86)	8 (14)	0	55.8 ± 11.3	36 (62)	
		*45 mg*	61	59 (97)	1 (1)	0	53.7 ± 11.9	35 (57)	
		*Placebo*	50	45 (90)	1 (2)	2 (4)	54 ± 8.8	29 (58)	
Wharton et al. 2025	Brazil, China, India, Japan, Korea, Slovakia, Spain, Taiwan, United States	*6 mg*	723	408 (57)	68 (9)	202 (28)	44.9 ± 12.1	469 (64.9)	72 weeks
		*12 mg*	725	405 (56)	60 (8)	201 (28)	45.4 ± 12.6	467 (64.4)	
		*36 mg*	730	394 (54)	67 (9)	214 (29)	44.9 ± 11.9	465 (63.7)	
		*Placebo*	949	539 (57)	72 (7)	267 (28)	45.1 ± 11.9	608 (64.1)	

*Note:* Italicized text indicates the different treatment arms in each study.

Abbreviation: NR, not reported.

### 3.3 Risk of Bias Assessment

**FIGURE 2:**
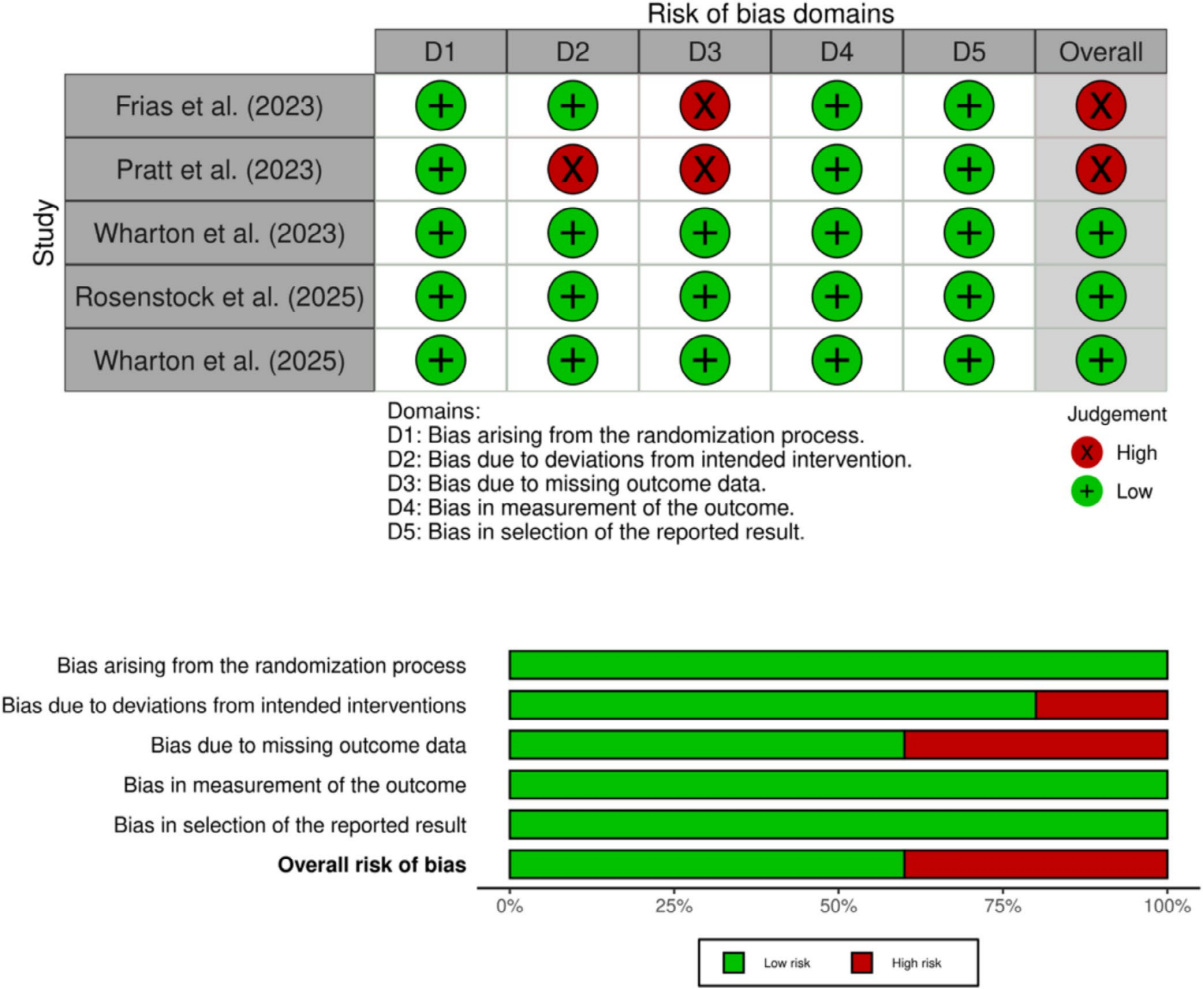
Summary of risk of bias of included studies.

Among the five trials, three were assessed to be of low risk of bias, while two studies were of high risk of bias (Frias et al. 2023 and Pratt et al. 2023). The main sources of bias were deviation from intended interventions (Pratt et al. 2023) and bias due to missing outcome data (Frias et al. 2023 and Pratt et al. 2023). The detailed quality evaluation of the selected studies is shown in Figure 2.

## 4 Efficacy Outcomes

### 4.1 Change in Waist Circumference (Cm)

**FIGURE 3:**
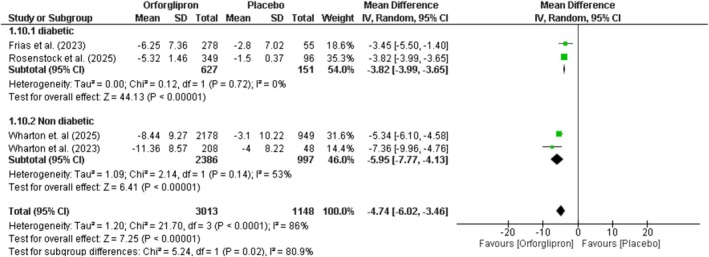
Effect of orforglipron versus placebo on waist circumference.

Orforglipron significantly reduced waist circumference compared to placebo by −4.74 cm (95% CI: −6.02 to −3.46; *p* < 0.00001; *I* ^2^ = 86%) (Figure 3). Subgroup analysis showed greater reductions in non‐diabetic patients (−5.95 cm; 95% CI: −7.77 to −4.13; *p* < 0.00001) compared to diabetic patients (−3.82 cm; 95% CI: −3.99 to −3.65; *p* < 00001), with significant between‐group differences (*p* = 0.02). Dose–response analysis revealed non‐significant effects at 3 mg (−1.57 cm; *p* = 0.27), but significant reductions at higher doses: 12 mg (−3.94 cm), 24 mg (−6.44 cm), 36 mg (−6.03 cm), and 45 mg (−6.9 cm), demonstrating a dose‐dependent effect (*p* = 0.008) (Figure S1).

### 4.2 Percentage Change in Body Weight

**FIGURE 4:**
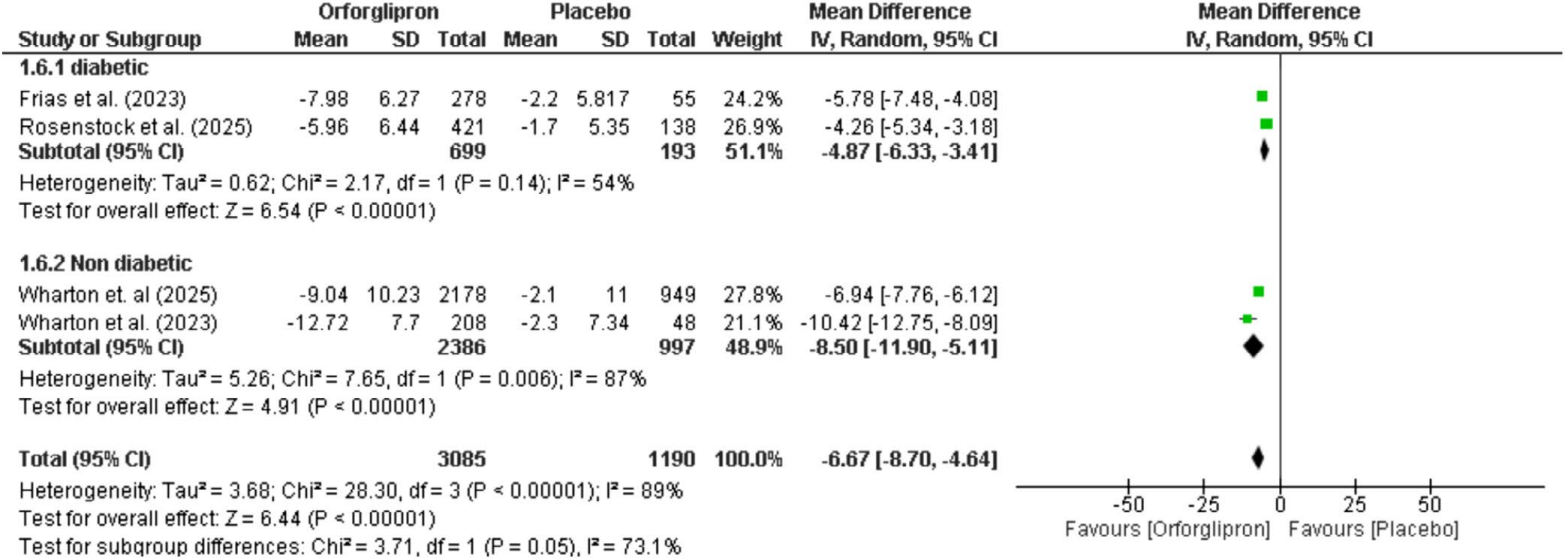
Effect of orforglipron versus placebo on body weight.

Orforglipron significantly reduced body weight compared to placebo by −6.67% (95% CI: −8.7 to −4.64; *p* < 0.00001; *I* ^2^ = 89%) (Figure 4). Subgroup analysis showed reductions of −4.87% (95% CI: −6.33 to −3.41; *p* < 0.00001) in diabetic patients and −8.5% (95% CI: −11.9 to −5.11; *p* < 0.00001) in non‐diabetic patients, with significant between‐group differences (*p* = 0.05). Dose–response analysis demonstrated significant reductions across all doses: 3 mg (−2.48%; 95% CI: −3.58 to −1.38), 12 mg (−5.36%; 95% CI: −6.78 to −3.93), 24 mg (−8.81%; 95% CI: −11.13 to −6.49), 36 mg (−8.24%; 95% CI: −10.28 to −6.2), and 45 mg (−9.8%; 95% CI: −14.7 to −4.91) (Figure S2).

### 4.3 Change in BMI (Kg/m^2^)

**FIGURE 5:**
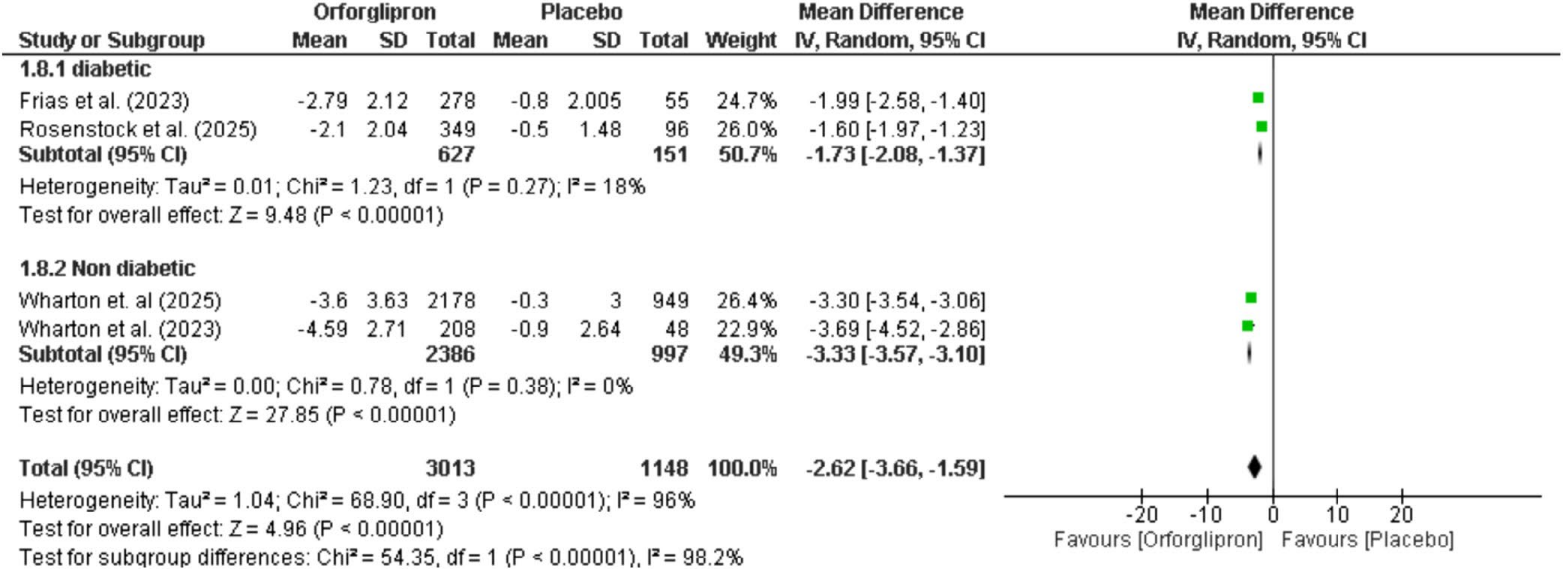
Effect of orforglipron versus placebo on BMI.

Orforglipron significantly reduced BMI compared to placebo by −2.62 kg/m^2^ (95% CI: −3.66 to −1.59; *p* < 0.00001; *I* ^2^ = 96%) (Figure 5). Subgroup analysis showed reductions of −1.73 kg/m^2^ (95% CI: −2.08 to −1.37; *p* < 0.00001) in diabetic obese patients and −3.33 kg/m^2^ (95% CI: −3.57 to −3.10) in non‐diabetic obese patients, with significant between‐group differences (*p* < 0.00001). Dose–response analysis demonstrated significant reductions across all doses: 3 mg (−0.89 kg/m^2^), 12 mg (−2.15 kg/m^2^), 24 mg (−3.15 kg/m^2^), 36 mg (−3.24 kg/m^2^), and 45 mg (−3.62 kg/m^2^) (Figure S3).

### 4.4 Change in HbA1c

**FIGURE 6:**
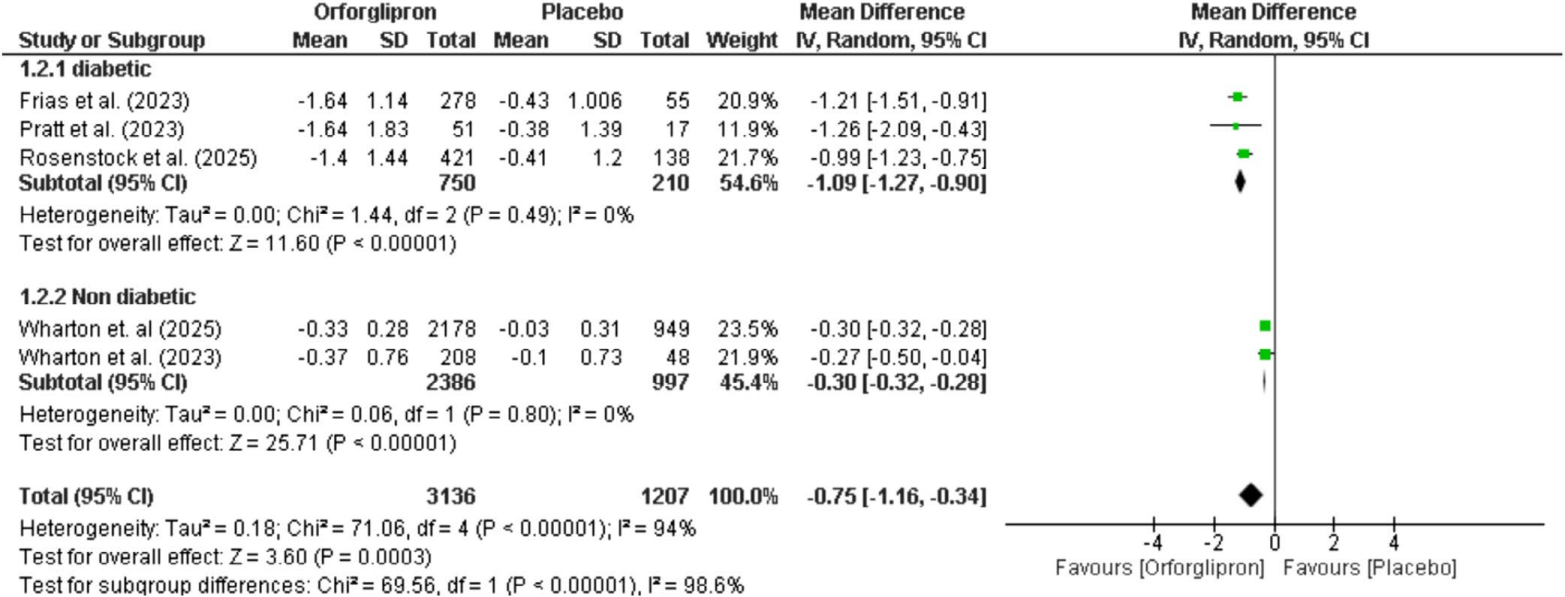
Effect of orforglipron versus placebo HbA1c.

Orforglipron significantly reduced HbA1c compared to placebo by −0.75% (95% CI: −1.16 to −0.34; *p* = 0.0003; *I* ^2^ = 94%) (Figure 6). Subgroup analysis showed a substantial reduction of −1.09% (95% CI: −1.27 to −0.9; *p* < 0.00001) in diabetic patients, while non‐diabetic patients showed a minimal reduction of −0.30% (95% CI: −0.32 to −0.28; *p* < 0.00001), with significant between‐group differences (*p* < 0.00001). Dose–response analysis revealed reductions across all doses: 3 mg (−0.81%), 12 mg (−0.76%), 24 mg (−0.82%), 36 mg (−0.81%), and 45 mg (−1.04%) (Figure S4).

### 4.5 Change in Fasting Serum Glucose in Mg/dL

Orforglipron significantly reduced fasting plasma glucose compared to placebo by −25.01 mg/dL (95% CI: −39.75 to −10.34; *p* = 0.0008; *I* ^2^ = 93%) (Figure S5a). Subgroup analysis showed a substantial reduction of −30.75 mg/dL (95% CI: −41.44 to −20.06; *p* < 0.00001) in diabetic patients, while non‐diabetic patients showed a smaller reduction of −8.84 mg/dL (95% CI: −9.74 to −7.94; *p* < 0.00001), with significant between‐group differences (*p* < 0.0001). Dose–response analysis revealed significant reductions at 3 mg (−20.22 mg/dL), 12 mg (−15.37 mg/dL), 24 mg (−52.20 mg/dL), 36 mg (−24.27 mg/dL), and 45 mg (−41.73 mg/dL) (Figure S5b).

### 4.6 Percentage Change in Total Serum Cholesterol

Orforglipron significantly reduced total serum cholesterol compared to placebo by −4.51 (95% CI: −6.91 to −2.11; *p* = 0.0002; *I* ^2^ = 47%) (Figure S6a). Subgroup analysis showed a substantial reduction of −6.56 (95% CI: −13.91 to 0.8; *p* = 0.08) in diabetic participants, while non‐diabetic participants showed a smaller reduction of −3.5 (95% CI: −9.74 to −7.94; *p* < 0.00001), with significant between‐group differences (*p* = 0.42). Dose–response analysis demonstrated significant reductions at 3 mg (−6.46), 12 mg (−4.11), 24 mg (−7.73), 36 mg (−5.19), and 45 mg (−7.98) (Figure S6b).

### 4.7 Percentage Change in LDL Cholesterol

Orforglipron significantly reduced LDL cholesterol compared to placebo by −5.34% (95% CI: −7.10 to −3.58; *p* < 0.00001; *I* ^2^ = 0%). Low heterogeneity (*I* ^2^ = 0%) was observed, indicating consistency across trials. Subgroup analysis showed reductions of −7.72% (95% CI: −14.85 to −0.58; *p* = 0.03) in diabetic patients and −5.12% (95% CI: −7 to −3.23; *p* < 0.00001) in non‐diabetic patients, with no significant between‐group differences (*p* = 0.49) (Figure S7a). Dose–response analysis revealed variable effects across doses: 3 mg (−10.27%), 12 mg (−6.15%), 24 mg (−9.34%), 36 mg (−5.34%), and 45 mg (−8.35%) (Figure S7b).

### 4.8 Percentage Change in HDL Cholesterol

Orforglipron significantly increased HDL cholesterol compared to placebo by 2.94% (95% CI: 1.38 to 4.49; *p* = 0.0002; *I* ^2^ = 20%). Subgroup analysis showed a smaller, non‐significant increase of 1.45% (95% CI: −2.49 to 5.39; *p* = 0.47) in diabetics, while non‐diabetics showed a significant increase of 3.55% (95% CI: 2.20 to 4.89; *p* < 0.00001), with no significant between‐group differences (*p* = 0.32) (Figure S8a). Dose–response analysis revealed variable effects: 3 mg (−0.59%), 12 mg (2.90%), 24 mg (1.86%), 36 mg (4.03%), and 45 mg (1.28%) (Figure S8b).

### 4.9 Percentage Change in Triglycerides Level

Orforglipron significantly reduced triglycerides compared to placebo by −10.07% (95% CI: −12.33 to −7.80; *p* < 0.00001; *I* ^2^ = 0%). Low heterogeneity (*I* ^2^ = 0%) was observed, indicating consistency across trials. Subgroup analysis showed reductions of −9.89% (95% CI: −15.99 to −3.79; *p* = 0.001) in diabetic patients and −10.09% (95% CI: −12.54 to −7.65; *p* < 0.00001) in non‐diabetic patients, with no significant between‐group differences (*p* = 0.95) (Figure S9a). Dose–response analysis revealed variable but generally consistent reductions: 3 mg (−8.30%), 12 mg (−10.66%), 24 mg (−13.60%), 36 mg (−15.91%), and 45 mg (−14.46%) (Figure S9b).

## 5 Safety Outcomes

### 5.1 Any Treatment Emergent Adverse Events

Compared to placebo, the occurrence of TEAE (treatment‐emergent adverse events) was significantly higher with orforglipron 12 mg (RR 1.08;95% CI: 1.04 to 1.13; *p* < 0.0001), 24 mg (RR 1.16; 95% CI: 1.00, 1.36; *p* = 0.05), 36 mg (RR 1.08; 95% CI: 1.03 to 1.13; *p* = 0.0009), and 45 mg (RR 1.35; 95% CI: 1.05 to 1.74; *p* = 0.02) daily doses, with the highest rates observed with orforglipron 45 mg. The 3 mg dose showed no significant increase in TEAEs (RR 1.01; 95% CI: 0.87 to 1.16; *p* = 0.92) (Table 2).

**TABLE 2:** The results of safety outcomes of orforglipron versus placebo.

Outcome variables	Orforglipron arm	No of participants (trials)	Pooled effect RR (95% CI)	*p*	*I* ^2^ (%)
Any TEAE	3 mg	387 (2 RCTs)	1.01 (0.87 to 1.16)	0.92	13
	12 mg	2158 (4 RCTs)	1.08 (1.04 to 1.13)	< 0.0001	0
	24 mg	205 (2 RCTs)	1.16 (1 to 1.36)	0.05	0
	36 mg	2179 (4 RCTs)	1.08 (1.03 to 1.13)	0.0009	5
	45 mg	255 (3 RCTs)	1.35 (1.05 to 1.74)	0.02	59
Serious adverse event	3 mg	387 (2 RCTs)	1.37 (0.56 to 3.35)	0.49	0
	12 mg	2058 (3 RCTs)	1.1 (0.75 to 1.62)	0.62	0
	24 mg	205 (2 RCTs)	2.27 (0.65 to 7.95)	0.2	0
	36 mg	2197 (4 RCTs)	0.81 (0.54 to 1.23)	0.33	0
	45 mg	255 (3 RCTs)	0.71 (0.15 to 3.34)	0.66	0
Adverse events leading to discontinuation	3 mg	387 (2 RCTs)	2.77 (1.01 to 7.57)	0.05	0
	12 mg	2158 (4 RCTs)	2.95 (1.97 to 4.43)	< 0.00001	0
	24 mg	205 (2 RCTs)	4.61 (1.6 to 13.33)	0.005	0
	36 mg	2179 (4 RCTs)	3.68 (2.48 to 5.44)	< 0.00001	0
	45 mg	255 (3 RCTs)	1.42 (0.25 to 8.23)	0.69	55
Death	3 mg	387 (2 RCTs)	1.05 (0.16 to 7.1)	0.96	0
	12 mg	2058 (3 RCTs)	0.81 (0.15 to 4.3)	0.81	0
	24 mg	102 (1 RCT)	0.39 (0.02 to 9.33)	0.56	—
	36 mg	2071 (3 RCTs)	0.35 (0.06 to 2.2)	0.26	0
	45 mg	144 (2 RCTs)	0.29 (0.01 to 7.02)	0.45	—
Nausea	3 mg	387 (2 RCTs)	5 (2.14 to 11.71)	0.0002	0
	12 mg	486 (3 RCTs)	6.24 (3.43 to 11.36)	< 0.00001	0
	24 mg	205 (2 RCTs)	5.98 (2.99 to 11.98)	< 0.00001	0
	36 mg	503 (3 RCTs)	5.47 (3 to 10)	< 0.00001	0
	45 mg	255 (3 RCTs)	4.06 (2.06 to 8)	< 0.0001	0
Vomiting	3 mg	387 (2 RCTs)	3.33 (0.93 to 11.92)	0.06	0
	12 mg	486 (3 RCTs)	5.26 (2.25 to 12.29)	0.0001	0
	24 mg	205 (2 RCTs)	6.97 (2.55 to 19.07)	0.0002	0
	36 mg	503 (3 RCTs)	6.41 (2.71 to 15.18)	< 0.0001	5
	45 mg	255 (3 RCTs)	7.25 (2.81 to 18.7)	< 0.0001	0
Constipation	3 mg	387 (2 RCTs)	2.96 (1.14 to 7.68)	0.03	4
	12 mg	486 (3 RCTs)	4.61 (2.3 to 9.26)	< 0.0001	0
	24 mg	205 (2 RCTs)	5.71 (2.06 to 15.77)	0.0008	0
	36 mg	503 (3 RCTs)	4.16 (2.07 to 8.38)	< 0.0001	0
	45 mg	229 (2 RCTs)	3.08 (1.06 to 8.94)	0.04	0
Diarrhoea	3 mg	387 (2 RCTs)	2.35 (1.36 to 4.08)	0.002	0
	12 mg	486 (3 RCTs)	3.01 (1.69 to 5.36)	0.0002	0
	24 mg	205 (2 RCTs)	2.9 (1.42 to 5.94)	0.004	0
	36 mg	503 (3 RCTs)	1.97 (0.86 to 4.55)	0.11	44
	45 mg	255 (3 RCTs)	2.55 (1.22 to 5.35)	0.01	16

Abbreviations: CI, confidence interval; RR, risk ratio.

### 5.2 Serious Adverse Event

Serious adverse events were comparable between orforglipron and placebo across all doses: 3 mg (RR 1.37; 95% CI: 0.56 to 3.35; *p* = 0.49), 12 mg (RR 1.10; 95% CI: 0.75 to 1.62; *p* = 0.62), 24 mg (RR 2.27; 95% CI: 0.65 to 7.95; *p* = 0.20), 36 mg (RR 0.81; 95% CI: 0.54 to 1.23; *p* = 0.33), and 45 mg (RR 0.71; 95% CI: 0.15 to 3.34; *p* = 0.66), with no significant subgroup differences (*p* = 0.48) (Table 2).

### 5.3 Adverse Events Leading to Discontinuation

Treatment discontinuation due to adverse events was significantly higher with orforglipron compared to placebo across multiple doses (except 45 mg): 3 mg (RR 2.77; 95% CI: 1.01 to 7.57; *p* = 0.05), 12 mg (RR 2.95; 95% CI: 1.97 to 4.43; *p* < 0.00001), 24 mg (RR 4.61; 95% CI: 1.60 to 13.33; *p* = 0.005), and 36 mg (RR 3.68; 95% CI: 2.48 to 5.44; *p* < 0.00001) (Table 2).

### 5.4 Death

Mortality events were rare and comparable between orforglipron and placebo across all doses: 3 mg (RR 1.05; 95% CI: 0.16 to 7.10; *p* = 0.96), 12 mg (RR 0.81; 95% CI: 0.15 to 4.30; *p* = 0.81), 24 mg (RR 0.39; 95% CI: 0.02 to 9.33; *p* = 0.56), 36 mg (RR 0.35; 95% CI: 0.06 to 2.20; *p* = 0.26), and 45 mg (RR 0.29; 95% CI: 0.01 to 7.02; *p* = 0.45) (Table 2).

### 5.5 Gastrointestinal Adverse Events

Compared to placebo, orforglipron was associated with a higher occurrence of nausea, vomiting, and constipation at all doses. A higher risk of diarrhoea was observed with all doses except 36 mg (Table 2).

### 5.6 Long Term Versus Short Term Treatment Duration

Subgroup analysis by treatment duration revealed that for body weight, reductions were numerically greater in trials > 28 weeks duration compared to ≤ 28 weeks, though subgroup differences were not statistically significant (*p* = 0.45). Similarly, BMI reduction showed a greater effect in longer trials (−2.84 vs. −1.99 kg/m^2^). In contrast, HbA1c reduction was significantly greater in shorter‐duration trials (−1.22% vs. −0.51%) (Figure S10).

### 5.7 Sensitivity Analysis

Sensitivity analyses demonstrated that excluding any single study did not substantially change the pooled results, confirming the robustness and reliability of our findings (Table S4).

## 6 Discussion

This study‐level meta‐analysis of five randomised controlled trials aimed to provide comprehensive evidence on the efficacy and safety of orforglipron, a novel non‐peptide oral GLP‐1 receptor agonist, in obese patients with or without type 2 diabetes mellitus (T2DM). Our pooled findings demonstrated that orforglipron induced significant and dose‐dependent reductions in waist circumference, body weight, BMI, HbA1c, fasting plasma glucose and lipid parameters, supporting its potential role in obesity and metabolic management. However, it was also associated with a higher risk of gastrointestinal adverse events and treatment discontinuation, particularly at higher doses.

Orforglipron achieved substantial reductions in body weight (−6.67%), BMI (−2.62 kg/m^2^) and waist circumference (−4.74 cm) compared to placebo. These reductions are lower than the effects seen with injectable GLP‐1 receptor agonists such as semaglutide (15%–17% weight loss) and tirzepatide (15%–22% weight loss) but clinically meaningful and comparable to those observed with other oral anti‐obesity agents [14, 29]. The dose–response relationship was observed in our analysis, with maximal effects at 45 mg daily (9.8% weight reduction). Notably, non‐diabetic patients demonstrated greater weight loss (−8.5%) compared to diabetic patients (−4.87%), a finding consistent with previous GLP‐1 agonist studies [29, 30, 31, 32]. The absence of significant duration‐based differences in weight reduction aligns with prior trials. In the STEP‐1 trial, semaglutide 2.4 mg achieved 14.9% weight loss at week 68, with only a modest additional 0.3% (−15.2%) reduction observed at week 104 in STEP‐5, despite nearly doubling treatment duration [29, 33].

The waist circumference reduction of −4.74 cm is particularly noteworthy, as central adiposity is strongly associated with cardiovascular risk, insulin resistance, and metabolic syndrome [34]. Studies have demonstrated that each 1 cm reduction in waist circumference is associated with a 2% decrease in cardiovascular disease risk [35]. This suggests that orforglipron may offer benefits beyond weight reduction, potentially through preferential targeting of visceral adipose tissue deposition. Recent phase 2 data [13] have further demonstrated favorable effects of orforglipron on multiple cardiovascular risk markers, including reductions in systolic blood pressure, LDL cholesterol, triglycerides, apolipoprotein B, and inflammatory biomarkers such as hsCRP and IL‐6. These findings support the potential role of orforglipron in improving cardiometabolic outcomes, consistent with the effects observed in large cardiovascular outcome trials of injectable GLP‐1 receptor agonists, including LEADER (liraglutide) [36], SUSTAIN 6 (semaglutide) [37], and REWIND (dulaglutide) [38], which have demonstrated significant reductions in major adverse cardiovascular events. Although long‐term cardiovascular outcome data for orforglipron are not yet available, future large‐scale investigations are expected to determine whether these improvements in metabolic and inflammatory parameters translate into sustained cardiovascular protection comparable to that of established GLP‐1 receptor agonists.

Orforglipron demonstrated significant HbA1c reduction (−0.75%) and fasting glucose lowering (−25.01 mg/dL) compared to placebo. As expected, these effects were substantially more pronounced in diabetic patients (HbA1c: −1.09%; fasting glucose: −30.75 mg/dL) compared to non‐diabetic individuals (HbA1c: −0.30%; fasting glucose: −8.84 mg/dL). The HbA1c reduction of approximately 1% in diabetic patients is clinically significant and meets established thresholds for meaningful glycemic improvement, as each 1% reduction in HbA1c is associated with a 37% reduction in microvascular complications [39].

The glycemic benefits of orforglipron are comparable to those reported with oral semaglutide (HbA1c reduction of 0.9%–1.4%) and other GLP‐1 receptor agonists [40]. The glucose‐lowering effects are mediated through multiple mechanisms, including enhanced glucose‐dependent insulin secretion, suppression of inappropriate glucagon secretion, delayed gastric emptying, and increased insulin sensitivity [41]. Interestingly, the dose–response relationship for HbA1c was relatively flat across doses (ranging from −0.76% to −1.04%), suggesting that even lower doses may be effective for glycemic control, while higher doses may be reserved for patients requiring greater weight loss. This observation has important clinical implications for dose titration strategies and allows for individualised treatment approaches based on patient‐specific goals.

Orforglipron produced favorable changes across multiple lipid parameters, including reductions in total cholesterol (−4.51%), LDL cholesterol (−5.34%), and triglycerides (−10.07%), alongside a modest increase in HDL cholesterol (2.94%). These lipid modifications are consistent with the known pleiotropic effects of GLP‐1 receptor agonists and may contribute to cardiovascular risk reduction beyond glucose control [42]. The 10% reduction in triglycerides is particularly impressive and consistent across doses. This magnitude of triglyceride reduction is comparable to that achieved with fibrates and may be mediated through improved insulin sensitivity, reduced hepatic lipogenesis, and enhanced lipid oxidation. Although elevated triglycerides are recognized as an independent cardiovascular risk factor, their reduction alone has not consistently translated into meaningful cardiovascular event reduction. For instance, lipid‐modifying agents such as fenofibrate and pemafibrate primarily lower triglycerides but have shown limited cardiovascular benefit, with only a non‐significant trend toward risk reduction in large outcome trials [43, 44]. Thus, the cardiovascular advantages of GLP‐1 receptor agonists are more plausibly explained by their broad metabolic effects, including improvements in glycemic control, body weight, blood pressure, and inflammation, rather than isolated triglyceride lowering. The LDL cholesterol reduction of 5.34% is modest but clinically relevant, as each 1 mmol/L (approximately 38.7 mg/dL) reduction in LDL cholesterol is associated with a 22% reduction in major vascular events [45].

The safety analysis revealed dose‐dependent increases in treatment‐emergent adverse events at doses ≥ 12 mg (RR 1.08–1.35), while 3 mg showed no significant increase (RR 1.01), suggesting this is an appropriate starting dose. Gastrointestinal adverse events (nausea, vomiting, diarrhoea, constipation) were increased across all doses, consistent with the GLP‐1 receptor agonist class effect reflecting delayed gastric emptying and altered gastrointestinal motility [46]. These symptoms typically peak with dose escalation and diminish with continued treatment; this pattern is consistent across all GLP‐1 receptor agonists [19, 47]. Discontinuation rates were significantly elevated (RR 2.77–4.61 across most doses), representing a clinically important limitation. Approximately 2–5 times more patients discontinued orforglipron versus placebo due to adverse events, comparable to other GLP‐1 receptor agonists where 5%–15% discontinue in clinical trials [48]. Real‐world discontinuation rates may be higher, emphasising the need for mitigation strategies including gradual dose titration, antiemetic prophylaxis, patient education, and dietary modifications. The oral route eliminates barriers associated with injectable therapies including needle phobia and injection site reactions.

### 6.1 Strengths and Limitations

This meta‐analysis represents the first comprehensive synthesis of randomised controlled trials evaluating the safety and efficacy of orforglipron in obese adults, with and without diabetes. In our study, the substantial heterogeneity was appropriately explored through subgroup analyses, revealing clinically meaningful differences between diabetic and non‐diabetic populations. Nevertheless, certain limitations should be acknowledged. The number of available RCTs was relatively small, and most trials were of short duration, limiting conclusions on long‐term safety and sustainability of efficacy. Significant heterogeneity was observed in some outcomes, likely reflecting differences in study populations, baseline characteristics, and intervention durations. Moreover, data conversions were performed using the Meta‐Converter tool. While this method allows for data harmonisation across studies, it introduces a degree of estimation that may have contributed to the high heterogeneity observed. Furthermore, all included trials were sponsored by the drug manufacturer, introducing a potential risk of sponsorship bias. An additional limitation is the reliance on aggregate data rather than individual patient‐level data (IPD), which limits the ability to account for confounders. Notably, data on gallstone formation and biliary adverse events were not systematically reported across the included trials. This represents a limitation of the current evidence, as rapid weight loss with GLP‐1 receptor agonists has been associated with an increased risk of cholelithiasis in prior studies [49]. Another limitation is that trial durations varied from 12 to 72 weeks. Subgroup analysis revealed no significant differences in weight reduction between shorter (≤ 28 weeks) and longer (> 28 weeks) trials (*p* = 0.21), despite greater numerical values for weight loss with longer treatment. However, HbA1c reduction was significantly greater in shorter trials (−1.22% vs. −0.51%; *p* = 0.004). This pattern is likely confounded by diabetic status as shorter trials predominantly enrolled diabetic patients (Frias 28 weeks, Pratt 12 weeks) while longer trials enrolled non‐diabetic patients (Wharton 40 and 72 weeks).

Future research should focus on (1) long‐term studies to evaluate the durability of therapeutic benefits and identify rare adverse events; (2) cardiovascular outcome trials to determine whether metabolic improvements translate into reduced cardiovascular morbidity and mortality, as observed with certain injectable GLP‐1 receptor agonists [35, 45]; (3) comparative effectiveness trials to assess relative efficacy and safety, particularly with SGLT‐2 inhibitors, to optimize outcomes; and (4) real‐world effectiveness studies.

### 6.2 Conclusion

Orforglipron, an oral GLP‐1 receptor agonist, shows promising efficacy in weight reduction, glycemic control, and lipid improvement. While gastrointestinal adverse events and discontinuation rates are elevated, serious adverse events and mortality remain comparable to placebo, indicating acceptable safety. The oral formulation may enhance patient uptake compared to injectables. Future extensive studies confirming long‐term efficacy and safety outcomes are warranted to confirm these observations and determine their applicability in routine practice.

## Author Contributions

R.K.P.: Conceptualization, methodology, software, data curation, writing – original draft preparation. M.J.: Data curation, writing – original draft preparation. A.M.: Visualisation, investigation, data curation. K.A.J.: Software, validation, data curation. M.N.: Data curation, manuscript writing. M.A.A.: Data curation, manuscript writing. A.B.A.: Writing – original draft preparation. S.A.: Visualisation, investigation, data curation. M.S.: Writing – reviewing and editing, supervision. M.I.B.: Validation, writing – reviewing and editing. S.I.: Writing – reviewing and editing. M.N.S.: Writing – reviewing and editing. H.A.D.: Data curation, writing – original draft preparation. W.A.: Data curation, manuscript writing. R.M.O.: Writing – reviewing and editing.

## Ethics Statement

This study is a secondary analysis of published data and did not require ethical approval.

## Consent

The authors have nothing to report.

## Conflicts of Interest

The authors declare no conflicts of interest.

## Supporting information


**Figure S1:** Dose–response effect of orforglipron versus placebo on waist circumference reduction. Forest plot showing mean differences in waist circumference change (cm) for orforglipron administered at doses of daily 3 mg, daily 12 mg, daily 24 mg, daily 36 mg, and daily 45 mg compared to placebo.
**Figure S2:** Dose–Response effect of orforglipron versus placebo on body weight. Forest plot showing mean % change in body weight for orforglipron administered at doses of daily 3 mg, daily 12 mg, daily 24 mg, daily 36 mg, and daily 45 mg compared to placebo.
**Figure S3:** Dose–response effect of orforglipron versus placebo on BMI. Forest plot showing mean differences in BMI (kg/m^2^) for orforglipron administered at doses of daily 3 mg, daily 12 mg, daily 24 mg, daily 36 mg, and daily 45 mg compared to placebo.
**Figure S4:** Dose–response effect of orforglipron versus placebo on HbA1c. Forest plot showing mean differences in HbA1c (%) for orforglipron administered at doses of daily 3 mg, daily 12 mg, daily 24 mg, daily 36 mg, and daily 45 mg compared to placebo.
**Figure S5:** Effect of orforglipron versus placebo on fasting serum glucose. (a) Subgroup analysis based on diabetic status. (b) Forest plot showing mean differences in Fasting serum glucose (mg/dL) for orforglipron administered at doses of daily 3 mg, daily 12 mg, daily 24 mg, daily 36 mg, and daily 45 mg compared to placebo.
**Figure S6:** Effect of orforglipron versus placebo on total serum cholesterol. (a) Subgroup analysis based on diabetic status. (b) Forest plot showing mean % change in total serum cholesterol for orforglipron administered at doses of daily 3 mg, daily 12 mg, daily 24 mg, daily 36 mg, and daily 45 mg compared to placebo.
**Figure S7:** Effect of orforglipron versus placebo on LDL‐C. (a) Subgroup analysis based on diabetic status. (b) Forest plot showing mean % change in LDL cholesterol for orforglipron administered at doses of daily 3 mg, daily 12 mg, daily 24 mg, daily 36 mg, and daily 45 mg compared to placebo.
**Figure S8:** Effect of orforglipron versus placebo on HDL‐C. (a) Subgroup analysis based on diabetic status. (b) Forest plot showing mean % change in HDL cholesterol for orforglipron administered at doses of daily 3 mg, daily 12 mg, daily 24 mg, daily 36 mg, and daily 45 mg compared to placebo.
**Figure S9:** Effect of orforglipron versus placebo on triglycerides level. (a) Subgroup analysis based on diabetic status. (b) Forest plot showing mean % change in Triglycerides for orforglipron administered at doses of daily 3 mg, daily 12 mg, daily 24 mg, daily 36 mg, and daily 45 mg compared to placebo.
**Figure S10:** Effect of orforglipron vs. placebo. Forest plots showing pooled mean differences between orforglipron and placebo, stratified by treatment duration (≤ 28 weeks vs. > 28 weeks): (a) Change in waist circumference (cm), (b) percentage change in body weight (%), (c) change in BMI (kg/m^2^), and (d) Change in HbA1c (%).
**Table S1:** PRISMA statement checklist.
**Table S2:** Search strategy.
**Table S3:** Baseline values across studies.
**Table S4:** Sensitivity analysis.

## Data Availability

Data sharing not applicable to this article as no datasets were generated or analysed during the current study.

## References

[1] A. A. Khaleghi , N. Salari , N. Darvishi , et al., “Global Prevalence of Obesity in the Older Adults: A Meta‐Analysis,” Public Health in Practice 9 (2025): 100585, 10.1016/j.puhip.2025.100585. 39902301 PMC11788860

[2] T. Omer , “The Causes of Obesity: An In‐Depth Review,” AOWMC 10 (2020): 90–94, 10.15406/aowmc.2020.10.00312.

[3] A. N. M. S. Islam , H. Sultana , Nazmul Hassan Refat Md , Z. Farhana , A. Abdulbasah Kamil , and M. Meshbahur Rahman , “The Global Burden of Overweight‐Obesity and Its Association With Economic Status, Benefiting From STEPs Survey of WHO Member States: A Meta‐Analysis,” Preventive Medicine Reports 46 (2024): 102882, 10.1016/j.pmedr.2024.102882. 39290257 PMC11406007

[4] D.‐T. Chu , N. T. Minh Nguyet , T. C. Dinh , et al., “An Update on Physical Health and Economic Consequences of Overweight and Obesity,” Diabetes and Metabolic Syndrome: Clinical Research and Reviews 12 (2018): 1095–1100, 10.1016/j.dsx.2018.05.004. 29799416

[5] S. Hildebrand and A. Pfeifer , “The Obesity Pandemic and Its Impact on Non‐Communicable Disease Burden,” Pflügers Archiv ‐ European Journal of Physiology 477 (2025): 657–668, 10.1007/s00424-025-03066-8. 39924587 PMC12003543

[6] A. Okunogbe , R. Nugent , G. Spencer , J. Powis , J. Ralston , and J. Wilding , “Economic Impacts of Overweight and Obesity: Current and Future Estimates for 161 Countries,” BMJ Global Health 7 (2022): e009773, 10.1136/bmjgh-2022-009773. PMC9494015 36130777

[7] N. J. Sweis , “The Economic Burden of Obesity in 2024: A Cost Analysis Using the Value of a Statistical Life,” Critical Public Health 34 (2024): 1–13, 10.1080/09581596.2024.2333407.40182057

[8] D. M. Williams , A. Nawaz , and M. Evans , “Drug Therapy in Obesity: A Review of Current and Emerging Treatments,” Diabetes Therapy 11 (2020): 1199–1216, 10.1007/s13300-020-00816-y. 32297119 PMC7261312

[9] J. S. Baker , R. Supriya , F. Dutheil , and Y. Gao , “Obesity: Treatments, Conceptualizations, and Future Directions for a Growing Problem,” Biology 11 (2022): 160, 10.3390/biology11020160. 35205027 PMC8869388

[10] M. M. Ashour , M. Mabrouk , M. A. Aboelnasr , H. H. Beherei , K. M. Tohamy , and D. B. Das , “Anti‐Obesity Drug Delivery Systems: Recent Progress and Challenges,” Pharmaceutics 15 (2023): 2635, 10.3390/pharmaceutics15112635. 38004612 PMC10674714

[11] M.‐S. Popoviciu , L. Păduraru , G. Yahya , K. Metwally , and S. Cavalu , “Emerging Role of GLP‐1 Agonists in Obesity: A Comprehensive Review of Randomised Controlled Trials,” International Journal of Molecular Sciences 24 (2023): 10449, 10.3390/ijms241310449. 37445623 PMC10341852

[12] W. Ghusn and M. D. Hurtado , “Glucagon‐Like Receptor‐1 Agonists for Obesity: Weight Loss Outcomes, Tolerability, Side Effects, and Risks,” Obesity Pillars 12 (2024): 100127, 10.1016/j.obpill.2024.100127. 39286601 PMC11404059

[13] S. Wharton , J. Rosenstock , M. Konige , et al., “Treatment With Orforglipron, an Oral Glucagon Like Peptide‐1 Receptor Agonist, Is Associated With Improvements of CV Risk Biomarkers in Participants With Type 2 Diabetes or Obesity Without Diabetes,” Cardiovascular Diabetology 24 (2025): 240, 10.1186/s12933-025-02781-x. 40481478 PMC12142847

[14] A. M. Jastreboff , L. J. Aronne , N. N. Ahmad , et al., “Tirzepatide Once Weekly for the Treatment of Obesity,” New England Journal of Medicine 387 (2022): 205–216, 10.1056/NEJMoa2206038. 35658024

[15] T. Weiss , L. Yang , R. D. Carr , et al., “Real‐World Weight Change, Adherence, and Discontinuation Among Patients With Type 2 Diabetes Initiating Glucagon‐Like Peptide‐1 Receptor Agonists in the UK,” BMJ Open Diabetes Research & Care 10 (2022): e002517, 10.1136/bmjdrc-2021-002517. PMC8804648 35101924

[16] S. T. Buckley , T. A. Bækdal , A. Vegge , et al., “Transcellular Stomach Absorption of a Derivatized Glucagon‐Like Peptide‐1 Receptor Agonist,” Science Translational Medicine 10 (2018): eaar7047, 10.1126/scitranslmed.aar7047. 30429357

[17] C. Solis‐Herrera , M. P. Kane , and C. Triplitt , “Current Understanding of Sodium N‐(8‐[2‐Hydroxylbenzoyl] Amino) Caprylate (SNAC) as an Absorption Enhancer: The Oral Semaglutide Experience,” Clinical Diabetes 42 (2024): 74–86, 10.2337/cd22-0118. 38230324 PMC10788673

[18] X. Ma , R. Liu , E. J. Pratt , C. T. Benson , S. N. Bhattachar , and K. W. Sloop , “Effect of Food Consumption on the Pharmacokinetics, Safety, and Tolerability of Once‐Daily Orally Administered Orforglipron (LY3502970), a Non‐Peptide GLP‐1 Receptor Agonist,” Diabetes Therapy 15 (2024): 819–832, 10.1007/s13300-024-01554-1. 38402332 PMC10951152

[19] J. P. Frias , S. Hsia , S. Eyde , et al., “Efficacy and Safety of Oral Orforglipron in Patients With Type 2 Diabetes: A Multicentre, Randomised, Dose‐Response, Phase 2 Study,” Lancet 402 (2023): 472–483, 10.1016/S0140-6736(23)01302-8. 37369232

[20] J. P. T. Higgins , J. Thomas , J. Chandler , et al., Cochrane Handbook for Systematic Reviews of Interventions, 1st ed. (Wiley, 2019), 10.1002/9781119536604.

[21] M. J. Page , J. E. McKenzie , P. M. Bossuyt , et al., “Statement: An Updated Guideline for Reporting Systematic Reviews,” BMJ 2021 (2020): n71, 10.1136/bmj.n71. PMC8005924 33782057

[22] “WebPlotDigitizer,” n.d., https://web.eecs.utk.edu/~dcostine/personal/PowerDeviceLib/DigiTest/index.html.

[23] J. A. C. Sterne , J. Savović , M. J. Page , et al., “RoB 2: A Revised Tool for Assessing Risk of Bias in Randomised Trials,” BMJ 366 (2019): l4898, 10.1136/bmj.l4898. 31462531

[24] A. Abbas , M. T. Hefnawy , and A. Negida , “Meta‐Analysis Accelerator: A Comprehensive Tool for Statistical Data Conversion in Systematic Reviews With Meta‐Analysis,” BMC Medical Research Methodology 24 (2024): 243, 10.1186/s12874-024-02356-6. 39425031 PMC11487830

[25] E. Pratt , X. Ma , R. Liu , et al., “Orforglipron (LY3502970), a Novel, Oral Non‐Peptide Glucagon‐Like Peptide‐1 Receptor Agonist: A Phase 1b, Multicentre, Blinded, Placebo‐Controlled, Randomized, Multiple‐Ascending‐Dose Study in People With Type 2 Diabetes,” Diabetes, Obesity & Metabolism 25 (2023): 2642–2649, 10.1111/dom.15150. 37264711

[26] J. Rosenstock , S. Hsia , L. Nevarez Ruiz , et al., “Orforglipron, an Oral Small‐Molecule GLP‐1 Receptor Agonist, in Early Type 2 Diabetes,” New England Journal of Medicine 393 (2025): 1065–1076, 10.1056/NEJMoa2505669. 40544435

[27] S. Wharton , T. Blevins , L. Connery , et al., “Daily Oral GLP‐1 Receptor Agonist Orforglipron for Adults With Obesity,” New England Journal of Medicine 389 (2023): 877–888, 10.1056/NEJMoa2302392. 37351564

[28] S. Wharton , L. J. Aronne , A. Stefanski , et al., “Orforglipron, an Oral Small‐Molecule GLP‐1 Receptor Agonist for Obesity Treatment,” New England Journal of Medicine 393 (2025): 1796–1806, 10.1056/NEJMoa2511774. 40960239

[29] J. P. H. Wilding , R. L. Batterham , S. Calanna , et al., “Once‐Weekly Semaglutide in Adults With Overweight or Obesity,” New England Journal of Medicine 384 (2021): 989–1002, 10.1056/NEJMoa2032183. 33567185

[30] M. J. Davies , R. Bergenstal , B. Bode , et al., “Efficacy of Liraglutide for Weight Loss Among Patients With Type 2 Diabetes: The SCALE Diabetes Randomized Clinical Trial,” JAMA 314 (2015): 687–699, 10.1001/jama.2015.9676. 26284720

[31] D. H. Ryan , I. Lingvay , J. Deanfield , et al., “Long‐Term Weight Loss Effects of Semaglutide in Obesity Without Diabetes in the SELECT Trial,” Nature Medicine 30 (2024): 2049–2057, 10.1038/s41591-024-02996-7. PMC11271387 38740993

[32] A. K. Singh , R. Singh , A. Singh , and A. Misra , “Efficacy and Safety of Oral Semaglutide in Type 2 Diabetes: A Systematic Review of Real‐World Evidence,” Diabetes and Metabolic Syndrome: Clinical Research and Reviews 18 (2024): 103024, 10.1016/j.dsx.2024.103024. 38718449

[33] W. T. Garvey , R. L. Batterham , M. Bhatta , et al., “Two‐Year Effects of Semaglutide in Adults With Overweight or Obesity: The STEP 5 Trial,” Nature Medicine 28 (2022): 2083–2091, 10.1038/s41591-022-02026-4. PMC9556320 36216945

[34] R. Ross , I. J. Neeland , S. Yamashita , et al., “Waist Circumference as a Vital Sign in Clinical Practice: A Consensus Statement From the IAS and ICCR Working Group on Visceral Obesity,” Nature Reviews. Endocrinology 16 (2020): 177–189, 10.1038/s41574-019-0310-7. PMC7027970 32020062

[35] “Pathophysiology of Human Visceral Obesity: An Update | Physiological Reviews | American Physiological Society,” n.d., https://journals.physiology.org/doi/full/10.1152/physrev.00033.2011?rfr_dat=cr_pub++0pubmed&url_ver=Z39.88‐2003&rfr_id=ori%3Arid%3Acrossref.org. 10.1152/physrev.00033.2011 23303913

[36] S. P. Marso , G. H. Daniels , K. Brown‐Frandsen , et al., “Liraglutide and Cardiovascular Outcomes in Type 2 Diabetes,” New England Journal of Medicine 375 (2016): 311–322, 10.1056/NEJMoa1603827. 27295427 PMC4985288

[37] S. P. Marso , S. C. Bain , A. Consoli , et al., “Semaglutide and Cardiovascular Outcomes in Patients With Type 2 Diabetes,” New England Journal of Medicine 375 (2016): 1834–1844, 10.1056/NEJMoa1607141. 27633186

[38] H. C. Gerstein , H. M. Colhoun , G. R. Dagenais , et al., “Dulaglutide and Cardiovascular Outcomes in Type 2 Diabetes (REWIND): A Double‐Blind, Randomised Placebo‐Controlled Trial,” Lancet 394 (2019): 121–130, 10.1016/S0140-6736(19)31149-3. 31189511

[39] I. M. Stratton , A. I. Adler , H. A. W. Neil , et al., “Association of Glycaemia With Macrovascular and Microvascular Complications of Type 2 Diabetes (UKPDS 35): Prospective Observational Study,” BMJ 321 (2000): 405–412, 10.1136/bmj.321.7258.405. 10938048 PMC27454

[40] V. R. Aroda , J. Rosenstock , Y. Terauchi , et al., “PIONEER 1: Randomized Clinical Trial of the Efficacy and Safety of Oral Semaglutide Monotherapy in Comparison With Placebo in Patients With Type 2 Diabetes,” Diabetes Care 42 (2019): 1724–1732, 10.2337/dc19-0749. 31186300

[41] D. J. Drucker , “Mechanisms of Action and Therapeutic Application of Glucagon‐Like Peptide‐1,” Cell Metabolism 27 (2018): 740–756, 10.1016/j.cmet.2018.03.001. 29617641

[42] “GLP‐1 Receptor Agonists in the Treatment of Type 2 Diabetes—State‐of‐the‐Art—ScienceDirect,” n.d., https://www.sciencedirect.com/science/article/pii/S2212877820301769. 10.1016/j.molmet.2020.101102 PMC8085572 33068776

[43] “Effects of Combination Lipid Therapy in Type 2 Diabetes Mellitus,” New England Journal of Medicine 362 (2010): 1563–1574, 10.1056/NEJMoa1001282. 20228404 PMC2879499

[44] A. Das Pradhan , R. J. Glynn , J.‐C. Fruchart , et al., “Triglyceride Lowering With Pemafibrate to Reduce Cardiovascular Risk,” New England Journal of Medicine 387 (2022): 1923–1934, 10.1056/NEJMoa2210645. 36342113

[45] Collaboration CTT and CTT , “Efficacy and Safety of More Intensive Lowering of LDL Cholesterol: A Meta‐Analysis of Data From 170 000 Participants in 26 Randomised Trials,” Lancet 376 (2010): 1670–1681, 10.1016/S0140-6736(10)61350-5. 21067804 PMC2988224

[46] J. J. Holst , “The Physiology of Glucagon‐Like Peptide 1,” Physiological Reviews 87 (2007): 1409–1439, 10.1152/physrev.00034.2006. 17928588

[47] M. A. Nauck , D. R. Quast , J. Wefers , and J. J. Meier , “GLP‐1 Receptor Agonists in the Treatment of Type 2 Diabetes – State‐Of‐The‐Art,” Molecular Metabolism 46 (2021): 101102, 10.1016/j.molmet.2020.101102. 33068776 PMC8085572

[48] L. Blonde , G. E. Umpierrez , S. S. Reddy , et al., “American Association of Clinical Endocrinology Clinical Practice Guideline: Developing a Diabetes Mellitus Comprehensive Care Plan—2022 Update,” Endocrine Practice 28 (2022): 923–1049, 10.1016/j.eprac.2022.08.002. 35963508 PMC10200071

[49] L. He , J. Wang , F. Ping , et al., “Association of Glucagon‐Like Peptide‐1 Receptor Agonist Use With Risk of Gallbladder and Biliary Diseases: A Systematic Review and Meta‐Analysis of Randomized Clinical Trials,” JAMA Internal Medicine 182 (2022): 513–519, 10.1001/jamainternmed.2022.0338. 35344001 PMC8961394

