# Supplementary material for: Efficacy and Safety of Orforglipron in Obese Adults With or Without Diabetes: A Systematic Review and Meta‐Analysis
Source: Endocrinol Diabetes Metab. 2025 Nov 26;8(6):e70134. doi: 10.1002/edm2.70134 (PMC12653021; doi:10.1002/edm2.70134)
Supplement: Supplementary file 1 — Figure S1: Dose–response effect of orforglipron versus placebo on waist circumference reduction. Forest plot showing mean differences in waist circumference change (cm) for orforglipron administered at doses of daily 3 mg, daily 12 mg, daily 24 mg, daily 36 mg, and daily 45 mg compared to placebo. Figure S2: Dose–Response effect of orforglipron versus placebo on body weight. Forest plot showing mean % change in body weight for orforglipron administered at doses of daily 3 mg, daily 12 mg, daily 24 mg, daily 36 mg, and daily 45 mg compared to placebo. Figure S3: Dose–response effect of orforglipron versus placebo on BMI. Forest plot showing mean differences in BMI (kg/m2) for orforglipron administered at doses of daily 3 mg, daily 12 mg, daily 24 mg, daily 36 mg, and daily 45 mg compared to placebo. Figure S4: Dose–response effect of orforglipron versus placebo on HbA1c. Forest plot showing mean differences in HbA1c (%) for orforglipron administered at doses of daily 3 mg, daily 12 mg, daily 24 mg, daily 36 mg, and daily 45 mg compared to placebo. Figure S5: Effect of orforglipron versus placebo on fasting serum glucose. (a) Subgroup analysis based on diabetic status. (b) Forest plot showing mean differences in Fasting serum glucose (mg/dL) for orforglipron administered at doses of daily 3 mg, daily 12 mg, daily 24 mg, daily 36 mg, and daily 45 mg compared to placebo. Figure S6: Effect of orforglipron versus placebo on total serum cholesterol. (a) Subgroup analysis based on diabetic status. (b) Forest plot showing mean % change in total serum cholesterol for orforglipron administered at doses of daily 3 mg, daily 12 mg, daily 24 mg, daily 36 mg, and daily 45 mg compared to placebo. Figure S7: Effect of orforglipron versus placebo on LDL‐C. (a) Subgroup analysis based on diabetic status. (b) Forest plot showing mean % change in LDL cholesterol for orforglipron administered at doses of daily 3 mg, daily 12 mg, daily 24 mg, daily 36 mg, and daily 45 mg compared to place [file EDM2-8-e70134-s001.docx]

**SUPPLEMENTARY FILE**

**Efficacy and Safety of Orforglipron in Obese Adults with or without Diabetes: A Systematic Review and Meta-Analysis**

**Supplementary Table S1:** PRISMA statement checklist

| **Section and Topic** | **Item #** | **Checklist item** | **Location where item is reported** |
| --- | --- | --- | --- |
| **TITLE** | | |  |
| Title | 1 | Identify the report as a systematic review. | Page 1 |
| **ABSTRACT** | | |  |
| Abstract | 2 | See the PRISMA 2020 for Abstracts checklist. | Page 2 |
| **INTRODUCTION** | | |  |
| Rationale | 3 | Describe the rationale for the review in the context of existing knowledge. | Page 4-5 |
| Objectives | 4 | Provide an explicit statement of the objective(s) or question(s) the review addresses. | Page 5-6 |
| **METHODS** | | |  |
| Eligibility criteria | 5 | Specify the inclusion and exclusion criteria for the review and how studies were grouped for the syntheses. | Page 7 |
| Information sources | 6 | Specify all databases, registers, websites, organisations, reference lists and other sources searched or consulted to identify studies. Specify the date when each source was last searched or consulted. | Page 7 |
| Search strategy | 7 | Present the full search strategies for all databases, registers and websites, including any filters and limits used. | Supplementary Table S1 |
| Selection process | 8 | Specify the methods used to decide whether a study met the inclusion criteria of the review, including how many reviewers screened each record and each report retrieved, whether they worked independently, and if applicable, details of automation tools used in the process. | Page 8 |
| Data collection process | 9 | Specify the methods used to collect data from reports, including how many reviewers collected data from each report, whether they worked independently, any processes for obtaining or confirming data from study investigators, and if applicable, details of automation tools used in the process. | Page 8 |
| Data items | 10a | List and define all outcomes for which data were sought. Specify whether all results that were compatible with each outcome domain in each study were sought (e.g. for all measures, time points, analyses), and if not, the methods used to decide which results to collect. | Page 8-9 |
|  | 10b | List and define all other variables for which data were sought (e.g. participant and intervention characteristics, funding sources). Describe any assumptions made about any missing or unclear information. | Page 8-9 |
| Study risk of bias assessment | 11 | Specify the methods used to assess risk of bias in the included studies, including details of the tool(s) used, how many reviewers assessed each study and whether they worked independently, and if applicable, details of automation tools used in the process. | Page 8 |
| Effect measures | 12 | Specify for each outcome the effect measure(s) (e.g. risk ratio, mean difference) used in the synthesis or presentation of results. | Page 9 |
| Synthesis methods | 13a | Describe the processes used to decide which studies were eligible for each synthesis (e.g. tabulating the study intervention characteristics and comparing against the planned groups for each synthesis (item #5)). | Table 1 |
|  | 13b | Describe any methods required to prepare the data for presentation or synthesis, such as handling of missing summary statistics, or data conversions. | N/A |
|  | 13c | Describe any methods used to tabulate or visually display results of individual studies and syntheses. | Page 9 |
|  | 13d | Describe any methods used to synthesize results and provide a rationale for the choice(s). If meta-analysis was performed, describe the model(s), method(s) to identify the presence and extent of statistical heterogeneity, and software package(s) used. | Page 9 |
|  | 13e | Describe any methods used to explore possible causes of heterogeneity among study results (e.g. subgroup analysis, meta-regression). | N/A |
|  | 13f | Describe any sensitivity analyses conducted to assess robustness of the synthesized results. | N/A |
| Reporting bias assessment | 14 | Describe any methods used to assess risk of bias due to missing results in a synthesis (arising from reporting biases). | Page 8 |
| Certainty assessment | 15 | Describe any methods used to assess certainty (or confidence) in the body of evidence for an outcome. | N/A |
| **RESULTS** | | |  |
| Study selection | 16a | Describe the results of the search and selection process, from the number of records identified in the search to the number of studies included in the review, ideally using a flow diagram. | Page 10 |
|  | 16b | Cite studies that might appear to meet the inclusion criteria, but which were excluded, and explain why they were excluded. | N/A |
| Study characteristics | 17 | Cite each included study and present its characteristics. | Page 11, table 1 |
| Risk of bias in studies | 18 | Present assessments of risk of bias for each included study. | Page 12-13 |
| Results of individual studies | 19 | For all outcomes, present, for each study: (a) summary statistics for each group (where appropriate) and (b) an effect estimate and its precision (e.g. confidence/credible interval), ideally using structured tables or plots. | Page 13 |
| Results of syntheses | 20a | For each synthesis, briefly summarise the characteristics and risk of bias among contributing studies. | Page 12-13 |
|  | 20b | Present results of all statistical syntheses conducted. If meta-analysis was done, present for each the summary estimate and its precision (e.g. confidence/credible interval) and measures of statistical heterogeneity. If comparing groups, describe the direction of the effect. | Page 13 |
|  | 20c | Present results of all investigations of possible causes of heterogeneity among study results. | N/A |
|  | 20d | Present results of all sensitivity analyses conducted to assess the robustness of the synthesized results. | N/A |
| Reporting biases | 21 | Present assessments of risk of bias due to missing results (arising from reporting biases) for each synthesis assessed. | Page 13 |
| Certainty of evidence | 22 | Present assessments of certainty (or confidence) in the body of evidence for each outcome assessed. | N/A |
| **DISCUSSION** | | |  |
| Discussion | 23a | Provide a general interpretation of the results in the context of other evidence. | Page 22 |
|  | 23b | Discuss any limitations of the evidence included in the review. | Page 24 |
|  | 23c | Discuss any limitations of the review processes used. | Page 24 |
|  | 23d | Discuss implications of the results for practice, policy, and future research. | Page 24-25 |
| **OTHER INFORMATION** | | |  |
| Registration and protocol | 24a | Provide registration information for the review, including register name and registration number, or state that the review was not registered. | Page 7 |
|  | 24b | Indicate where the review protocol can be accessed, or state that a protocol was not prepared. | - |
|  | 24c | Describe and explain any amendments to information provided at registration or in the protocol. | - |
| Support | 25 | Describe sources of financial or non-financial support for the review, and the role of the funders or sponsors in the review. | Page 26 |
| Competing interests | 26 | Declare any competing interests of review authors. | Page 26 |
| Availability of data, code and other materials | 27 | Report which of the following are publicly available and where they can be found: template data collection forms; data extracted from included studies; data used for all analyses; analytic code; any other materials used in the review. | - |

**Supplementary Table S2:** Search strategy

| **P** | **I** | **C** | **O** |
| --- | --- | --- | --- |
| "Obesity"[Mesh] | "orforglipron" [Supplementary Concept] |  |  |
|  | LY3502970 |  |  |

**PUBMED: 51 Results
19/10/2025**
("orforglipron" [Supplementary Concept]) OR (LY3502970)

**CHOCHRANE:68
19/10/2025**

Date Run: 19/10/2025

Comment:

| ID | Search | Hits |
| --- | --- | --- |
| #1 | orforglipron | 49 |
| #2 | LY3502970 | 51 |
| #3 | #1 OR #2 | 65 |

**Clinical Trials.gov 44 results 19/10/2025**

Condition/disease

Other terms

Intervention/treatment: orforglipron

**EMBASE: 170**

**19/10/2025**

'orforglipron'/exp OR '3 [1 [2 [ [2 (4 fluoro 3, 5 dimethylphenyl) 3 [3 (4 fluoro 1 methyl 1h indazol 5 yl) 2, 3 dihydro 2 oxo 1h imidazol 1 yl] 2, 4, 6, 7 tetrahydro 4 methyl 5h pyrazolo [4, 3 c] pyridin 5 yl] carbonyl] 5 [tetrahydro 2, 2 dimethyl 2h pyran 4 yl] 1h indol 1 yl] 2 methylcyclopropyl] 1, 2, 4 oxadiazol 5 (2h) one' OR '3 [1 [2 [ [2 (4 fluoro 3, 5 dimethylphenyl) 3 [3 (4 fluoro 1 methylindazol 5 yl) 2, 3 dihydro 2 oxoimidazol 1 yl] 2, 4, 6, 7 tetrahydro 4 methylpyrazolo [4, 3 c] pyridin 5 yl] carbonyl] 5 [tetrahydro 2, 2 dimethylpyran 4 yl] indol 1 yl] 2 methylcyclopropyl] 1, 2, 4 oxadiazol 5 one' OR '3 [1 [5 [2, 2 dimethyl 4 oxanyl] 2 [2 (4 fluoro 3, 5 dimethylbenzene) 3 [3 (4 fluoro 1 methyl 5 indazolyl) 2 oxo 1 imidazolyl] 4 methyl 6, 7 dihydro 4h pyrazolo [4, 3 c] pyridine 5 carbonyl] 1 indolyl] 2 methylcyclopropyl] 1, 2, 4 oxa 5 diazolidinone' OR '3 [1 [5 [2, 2 dimethyl 4 oxanyl] 2 [2 (4 fluoro 3, 5 dimethylphenyl) 3 [3 (4 fluoro 1 methyl 5 indazolyl) 2 oxo 1 imidazolyl] 4 methyl 6, 7 dihydro 4h pyrazolo [4, 3 c] pyridine 5 carbonyl] 1 indolyl] 2 methylcyclopropyl] 1, 2, 4 oxa 5 diazolidinone' OR '3 [1 [5 [2, 2 dimethyloxan 4 yl] 2 [2 (4 fluoro 3, 5 dimethylbenzene) 3 [3 (4 fluoro 1 methylindazol 5 yl) 2 oxoimidazol 1 yl] 4 methyl 6, 7 dihydro 4h pyrazolo [4, 3 c] pyridine 5 carbonyl] indol 1 yl] 2 methylcyclopropyl] 1, 2, 4 oxadiazolidin 5 one' OR '3 [1 [5 [2, 2 dimethyloxan 4 yl] 2 [2 (4 fluoro 3, 5 dimethylphenyl) 3 [3 (4 fluoro 1 methylindazol 5 yl) 2 oxoimidazol 1 yl] 4 methyl 6, 7 dihydro 4h pyrazolo [4, 3 c] pyridine 5 carbonyl] indol 1 yl] 2 methylcyclopropyl] 1, 2, 4 oxadiazolidin 5 one' OR '5 (5) [2, 2 dimethyloxan 4 yl] 1 (4) fluoro 3 (2) (4 fluoro 3, 5 dimethylphenyl) 1 (1), 3 (4), 6 (2) trimethyl 3 (2), 3 (4), 3 (6), 3 (7) tetrahydro 1 (1) h, 2 (2) h 3 (3, 5) pyrazolo [4, 3 c] pyridina 1 (5) indazola 5 (2, 1) indola 7 (3) [1, 2, 4] oxadiazola 2 (1, 3) imidazola 6 (1, 1) cyclopropanaheptaphane 2 (2), 4, 7 (5) (7(2) h) trione' OR 'ly 3502970' OR 'ly3502970' OR 'orforglipron' OR 'orforglipron calcium' OR 'owl 833' OR 'owl833'

**Supplementary Table S3:** Baseline values across studies

| **Study ID** | **Dosage (mg)** | **BMI, kg/m2,**  **(Mean±SD)** | **Weight kg, (Mean±SD)** | **HbA1C, %, (mean±SD)** | **Fasting serum glucose, mg/dL (Mean± SD)** | **Metformin use**  **n (%)** | **Duration of diabetes, years (Mean ± SD)** | **Waist circumference, cm (Mean ± SD)** |
| --- | --- | --- | --- | --- | --- | --- | --- | --- |
| **Frias et al. 2023** | **3mg** | 35.3± 8.2 | 99.3±25.4 | 8.0(0.8) | 164.0(40.9) | 44(86%) | 6.60(6.85) | 112.9(18.4) |
|  | **12mg** | 34.8± 6.3 | 99.3±18.1 | 8.2(0.9) | 172.1(42.8) | 52(93%) | 7.80(6.78) | 113.7(11.8) |
|  | **24mg** | 34.1±7.7 | 98.5±22.9 | 8.2(0.9) | 171.7(44.4) | 46(98%) | 6.37(5.02) | 113.2(15.3) |
|  | **36mg** | 34.4± 5.4 | 98.9±17.5 | 8.0(0.7) | 157.9(28.7) | 54(89%) | 6.1(4.71) | 112.1(12.7) |
|  | **45mg** | 36.4±6.9 | 104.6± 25.1 | 8.1(0.9) | 166.4(35.0) | 56(89%) | 6.7(5.86) | 116.0(16.6) |
|  | **placebo** | 35.8±6.2 | 102±18.8 | 8.1(0.9) | 172.0(42.9) | 51(93%) | 8.0(6.47) | 115.0(12.4) |
| **Rosenstock et al. 2025** | **3mg** | 32.9±8.0 | 90.3±25.7 | 7.93±0.86 | 142.9±38.7 | N/A | 4.0±4.8 | 107.0±16.5 |
|  | **12mg** | 33.3±7.8 | 90.6±23.1 | 7.98±0.91 | 155.3±55.1 | N/A | 5.1±6.0 | 107.7±16.9 |
|  | **36mg** | 33.1±7.3 | 90.1±22.9 | 8.07±0.90 | 148.8±40.0 | N/A | 4.2±5.1 | 107.6±17.0 |
|  | **placebo** | 32.9±6.8 | 90±20.7 | 7.96±0.89 | 143.3±42.2 | N/A | 4.4±5.6 | 106.9±14.1 |
| **Pratt et al. 2023** | **9mg** | 30.14±3.6 | 85.61±12.76 | 8.02 ± 0.62 | N/A | 7 (77.8) | 13.48 ± 8.29 | N/A |
|  | **15mg** | 30.39±3.61 | 88.02±14.36 | 7.84 ± 0.74 | N/A | 8 (80.0) | 15.02 ± 11.97 | N/A |
|  | **21mg** | 32.60±5.48 | 92.09±18.78 | 8.36 ± 1.31 | N/A | 14 (100) | 9.48 ± 5.48 | N/A |
|  | **27mg** | 30.62±3.55 | 92.80±15.36 | 7.82 ± 0.69 | N/A | 8 (88.9) | 7.60 ± 4.39 | N/A |
|  | **45mg** | 29.82±2.84 | 81.49±10.24 | 7.93 ± 0.79 | N/A | 9 (100.0) | 10.38 ± 4.78 | N/A |
|  | **placebo** | 31.31±4.86 | 90.29±20.04 | 8.09 ± 0.75 | N/A | 15 (88.2) | 8.63 ± 4.89 | N/A |
| **Wharton et al. 2023** | **12mg** | 37.7±7.7 | 107.5±25.3 | 5.5±0.4 | 94.4±9.8 | N/A | N/A | 114.4±16.5 |
|  | **24mg** | 38.1±7.7 | 112.1±30.2 | 5.7±0.3 | 97.5±12 | N/A | N/A | 120.1±19.1 |
|  | **36mg** | 38±6.3 | 108.3±25.4 | 5.6±0.4 | 96.8±13.3 | N/A | N/A | 117.3±15.4 |
|  | **45mg** | 37.7±6.6 | 108±24.4 | 5.6±0.3 | 95.2±9.7 | N/A | N/A | 116.9±13.7 |
|  | **placebo** | 37.8±6.5 | 107.6±25.2 | 5.6±0.4 | 97.2±10.2 | N/A | N/A | 115.5±15.4 |
| **Wharton et al. 2025** | **6 mg** | 37.0±6.5 | 103.2±21.7 | 5.6 ± 0.4 | 5.1±0.6 | N/A | N/A | 112.2±14.1 |
|  | **12 mg** | 36.7±6.5 | 102.2±21.6 | 5.6 ± 0.3 | 5.1 ±0.6 | N/A | N/A | 112.0±14.2 |
|  | **36 mg** | 36.9±6.7 | 103.1±23.2 | 5.6 ± 0.3 | 5.2±0.6 | N/A | N/A | 112.4±15.3 |
|  | **Placebo** | 37.1±6.3 | 103.9±22.0 | 5.6 ± 0.3 | 5.1±0.6 | N/A | N/A | 112.8±14.5 |

**Supplementary Table S4:** Sensitivity analysis

| **Orforglipron Vs. Placebo** | | | | |
| --- | --- | --- | --- | --- |
| **Outcome** | **Waist circumference, cm** | | | |
| **Omitted Study** | **Pooled MD** | **95% CI** | **P value** | **I^2^ value** |
| Frias et al. (2023) | -5.08 | -6.60 to -3.56 | < 0.00001 | 91% |
| Rosenstock et al. (2025) | -5.25 | -6.93 to -3.58 | < 0.00001 | 64% |
| Wharton et. al (2025) | -4.55 | -6.35 to -2.76 | < 0.00001 | 72% |
| Wharton et al. (2023) | -4.31 | -5.53 to -3.09 | < 0.00001 | 87% |
| **Outcome** | **Percentage Change in Body Weight** | | | |
| **Omitted Study** | **Pooled MD** | **95% CI** | **P value** | **I^2^ value** |
| Frias et al. (2023) | -7.01 | -9.69 to -4.33 | < 0.00001 | 93% |
| Rosenstock et al. (2025) | -7.51 | -9.56 to -5.47 | < 0.00001 | 81% |
| Wharton et. al (2025) | -6.68 | -9.86 to -3.49 | < 0.0001 | 91% |
| Wharton et al. (2023) | -5.68 | -7.49 to -3.86 | < 0.00001 | 87% |
| **Outcome** | **Change in BMI (kg/m^2^)** | | | |
| **Omitted Study** | **Pooled MD** | **95% CI** | **P value** | **I^2^ value** |
| Frias et al. (2023) | -2.84 | -4.14 to -1.54 | <0.0001 | 97% |
| Rosenstock et al. (2025) | -2.98 | -3.90 to -2.06 | <0.00001 | 89% |
| Wharton et. al (2025) | -2.37 | -3.42 to -1.32 | <0.0001 | 90% |
| Wharton et al. (2023) | -2.31 | -3.52 to -1.09 | 0.0002 | 97% |
| **Outcome** | **Change in HbA1c** | | | |
| **Omitted Study** | **Pooled MD** | **95% CI** | **P value** | **I^2^ value** |
| Frias et al. (2023) | -0.60 | -0.98 to -0.23 | 0.002 | 92% |
| Pratt et al. (2023) | -0.68 | -1.11 to -0.25 | 0.002 | 95% |
| Rosenstock et al. (2025) | -0.67 | -1.11 to -0.24 | 0.002 | 93% |
| Wharton et. al (2025) | -0.89 | -1.40 to -0.38 | 0.0006 | 90% |
| Wharton et al. (2023) | -0.90 | -1.47 to -0.33 | 0.002 | 96% |
| **Outcome** | **Change in Fasting Serum Glucose, mg/dL** | | | |
| **Omitted Study** | **Pooled MD** | **95% CI** | **P value** | **I^2^ value** |
| Frias et al. (2023) | -20.23 | -34.07 to -6.40 | 0.004 | 91% |
| Pratt et al. (2023) | -22.66 | -38.76 to -6.55 | 0.006 | 94% |
| Rosenstock et al. (2025) | -26.37 | -49.19 to -3.55 | 0.02 | 94% |
| Wharton et. al (2025) | -30.75 | -41.44 to -20.06 | <0.00001 | 60% |
| **Outcome** | **Percentage change in** **total serum cholesterol** | | | |
| **Omitted Study** | **Pooled MD** | **95% CI** | **P value** | **I^2^ value** |
| Frias et al. (2023) | -3.47 | -4.62 to -2.33 | <0.00001 | 0% |
| Rosenstock et al. (2025) | -5.64 | -9.66 to -1.63 | 0.006 | 64% |
| Wharton et. al (2025) | -5.84 | -9.99 to -1.69 | 0.006 | 54% |
| Wharton et al. (2023) | -4.59 | -7.65 to -1.53 | 0.003 | 62% |
| **Outcome** | **Percentage Change in L****DL Cholesterol** | | | |
| **Omitted Study** | **Pooled MD** | **95% CI** | **P value** | **I^2^ value** |
| Frias et al. (2023) | -5.14 | -6.92 to -3.36 | <0.00001 | 0% |
| Rosenstock et al. (2025) | -5.52 | -7.89 to -3.15 | <0.00001 | 5% |
| Wharton et. al (2025) | -6.78 | -10.95 to -2.61 | 0.001 | 0% |
| Wharton et al. (2023) | -5.28 | -7.09 to -3.47 | <0.00001 | 0% |
| **Outcome** | **Percentage Change in HDL Cholesterol** | | | |
| **Omitted Study** | **Pooled MD** | **95% CI** | **P value** | **I^2^ value** |
| Frias et al. (2023) | 3.50 | 2.25 to 4.75 | <0.00001 | 0% |
| Rosenstock et al. (2025) | 2.49 | 0.07 to 4.91 | 0.04 | 46% |
| Wharton et. al (2025) | 2.01 | -0.37 to 4.39 | 0.10 | 14% |
| Wharton et al. (2023) | 2.67 | 0.45 to 4.90 | 0.02 | 46% |
| **Outcome** | **Percentage Change in Triglycerides Level** | | | |
| **Omitted Study** | **Pooled MD** | **95% CI** | **P value** | **I^2^ value** |
| Frias et al. (2023) | -9.91 | -12.23 to -7.60 | <0.00001 | 0% |
| Rosenstock et al. (2025) | -10.26 | -12.65 to -7.86 | <0.00001 | 0% |
| Wharton et. al (2025) | -10.36 | -15.71 to -5.01 | 0.0001 | 0% |
| Wharton et al. (2023) | -9.98 | -12.30 to -7.67 | <0.00001 | 0% |

**Supplementary Figure S1:** Dose-Response Effect of Orforglipron vs Placebo on Waist Circumference Reduction


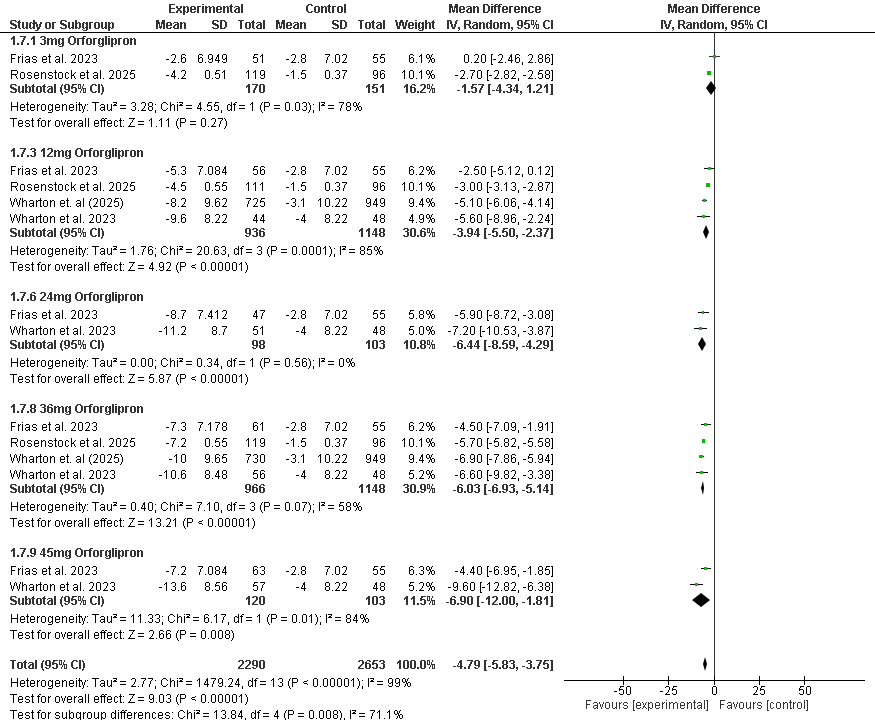


Forest plot showing mean differences in waist circumference change (cm) for orforglipron administered at doses of daily 3mg, daily 12mg, daily 24mg, daily 36mg, and daily 45mg compared to placebo.

**Supplementary Figure S2:** Dose-Response Effect of Orforglipron vs Placebo on body weight

**
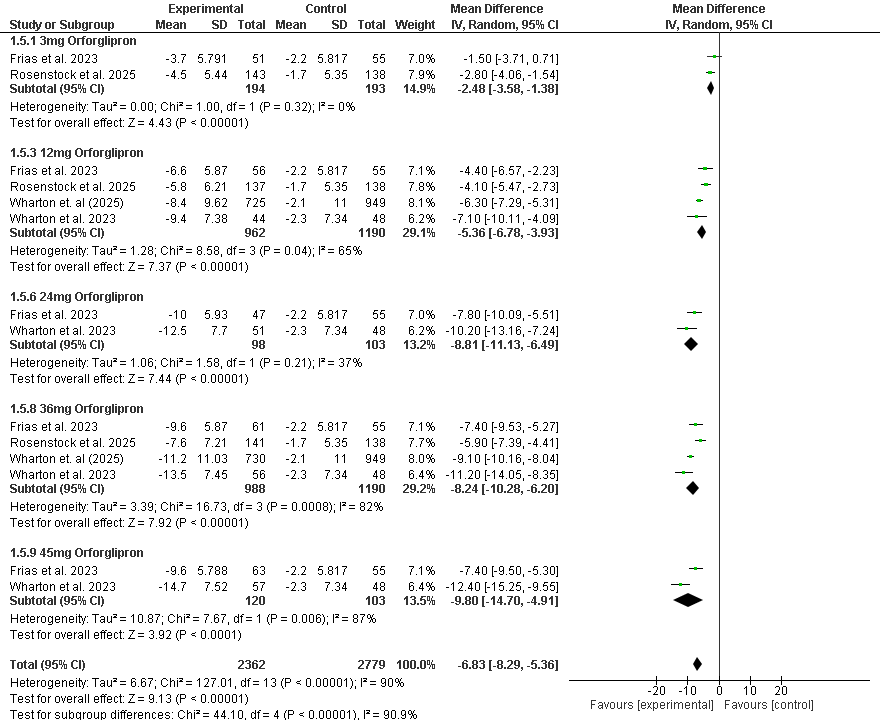
**

Forest plot showing mean % change in body weight for orforglipron administered at doses of daily 3mg, daily 12mg, daily 24mg, daily 36mg, and daily 45mg compared to placebo.

**Supplementary Figure S3:** Dose-Response Effect of Orforglipron vs Placebo on BMI


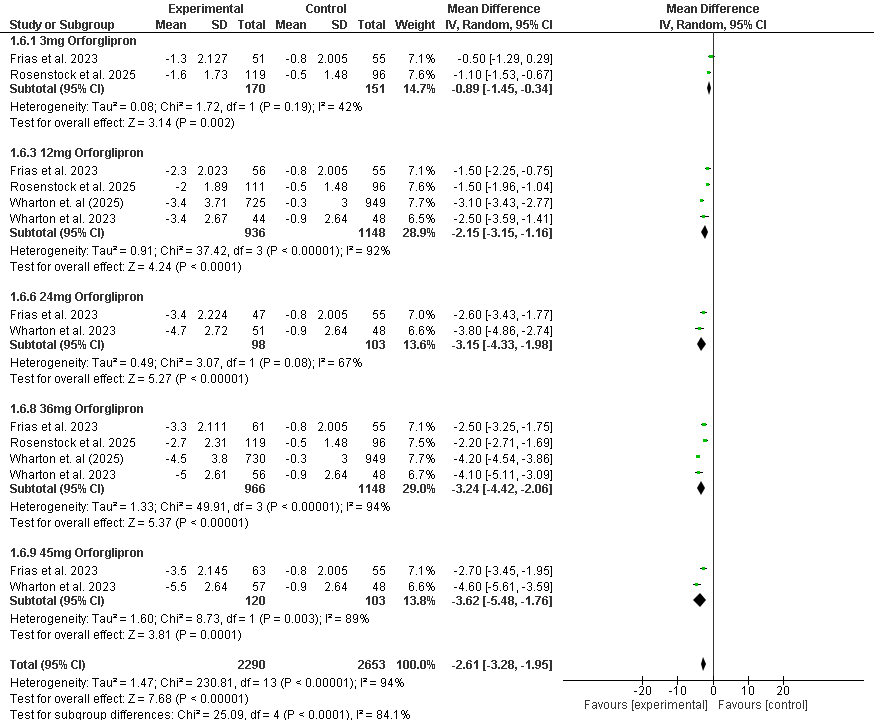


Forest plot showing mean differences in BMI (kg/m^2^) for orforglipron administered at doses of daily 3mg, daily 12mg, daily 24mg, daily 36mg, and daily 45mg compared to placebo.

**Supplementary Figure S4:** Dose-Response Effect of Orforglipron vs Placebo on HbA1c


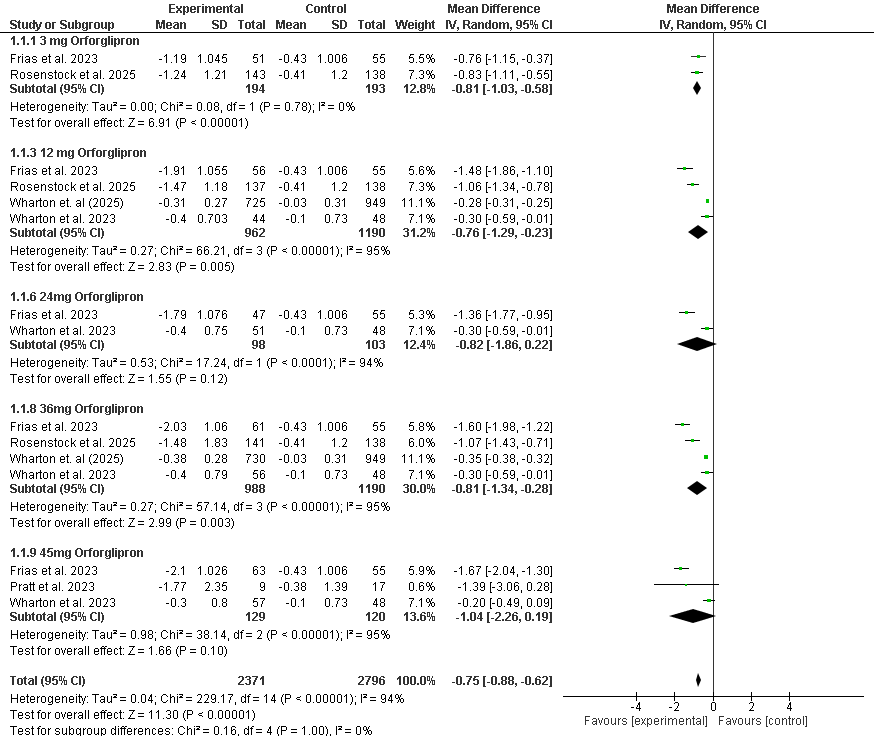


Forest plot showing mean differences in HbA1c (%) for orforglipron administered at doses of daily 3mg, daily 12mg,daily 24mg, daily 36mg, and daily 45mg compared to placebo.

**Supplementary Figure S5:** Effect of Orforglipron vs Placebo on Fasting serum glucose

**
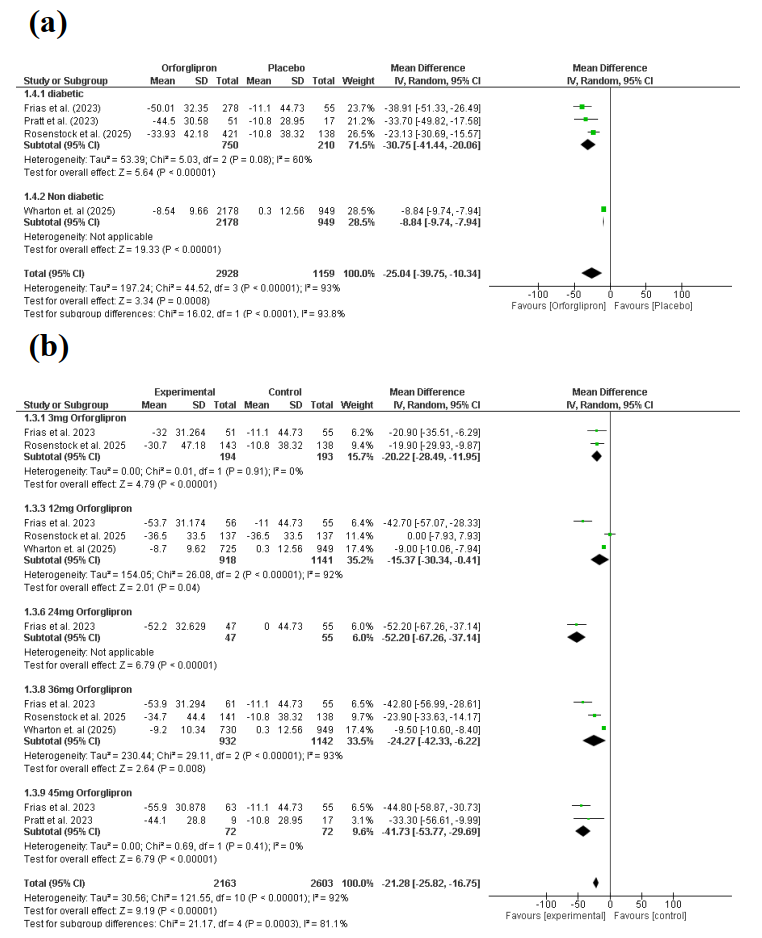
**

**(a)** Subgroup analysis based on diabetic status

**(b)** Forest plot showing mean differences in Fasting serum glucose (mg/dL) for orforglipron administered at doses of daily 3mg, daily 12mg, daily 24mg, daily 36mg, and daily 45mg compared to placebo.

**Supplementary Figure S6:** Effect of Orforglipron vs Placebo on total serum cholesterol
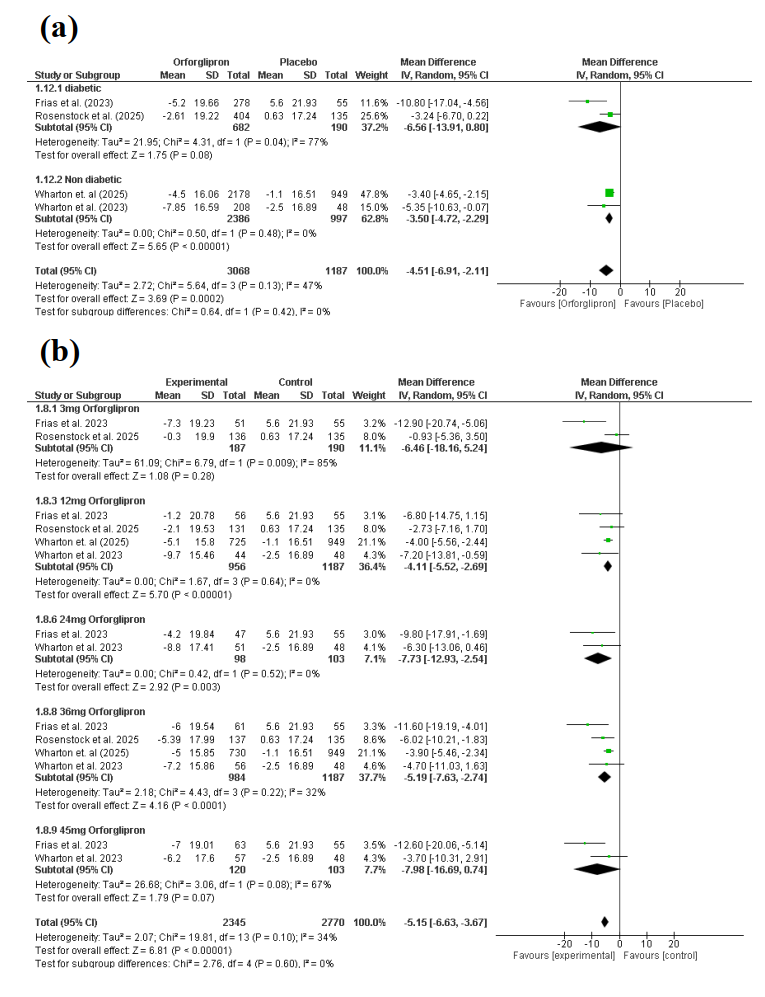


**(a)** Subgroup analysis based on diabetic status

**(b)** Forest plot showing mean % change in total serum cholesterol for orforglipron administered at doses of daily 3mg, daily 12mg, daily 24mg, daily 36mg, and daily 45mg compared to placebo.

**Supplementary Figure S7:** Effect of Orforglipron vs Placebo on LDL-C


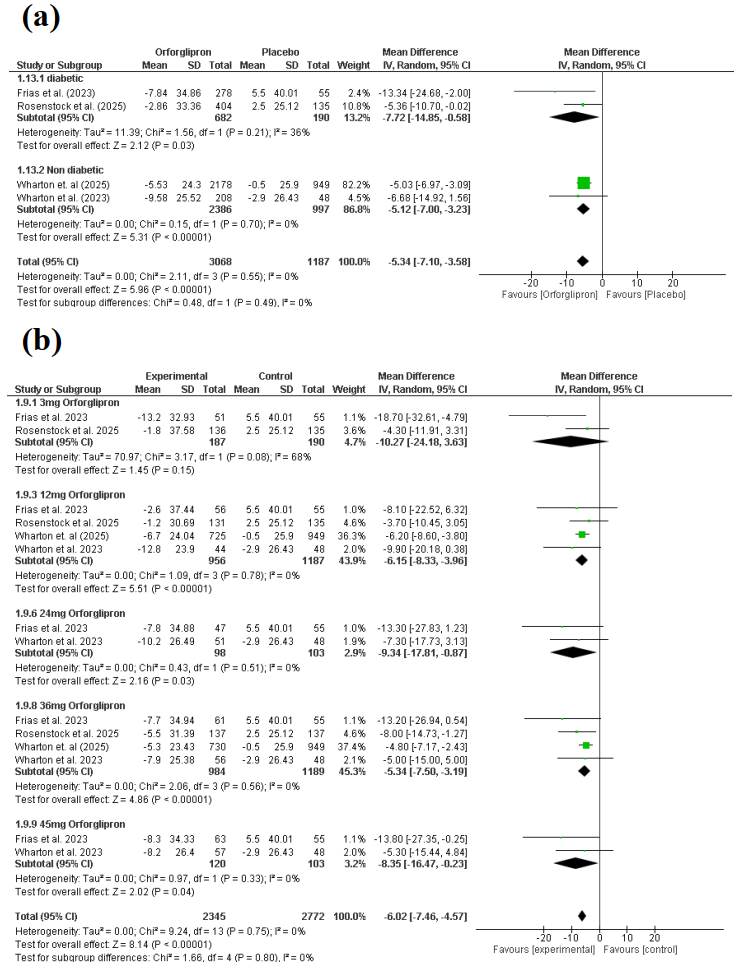


**(a)** Subgroup analysis based on diabetic status

**(b)** Forest plot showing mean % change in LDL cholesterol for orforglipron administered at doses of daily 3mg, daily 12mg, daily 24mg, daily 36mg, and daily 45mg compared to placebo.

**Supplementary Figure S8:** Effect of Orforglipron vs Placebo on HDL-C


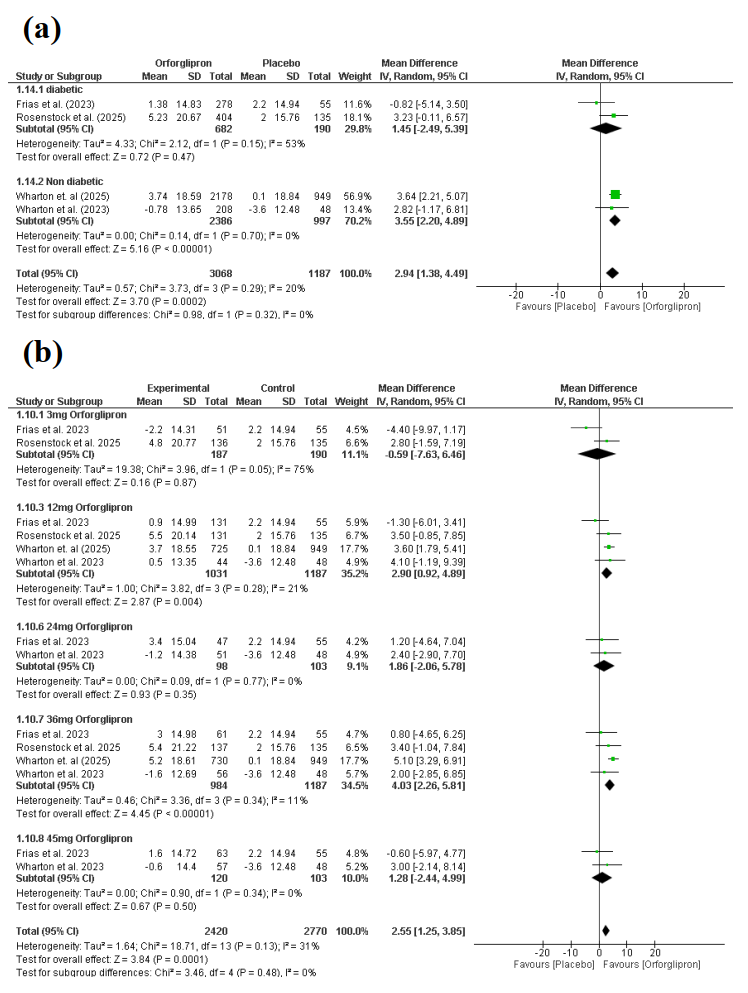


**(a)** Subgroup analysis based on diabetic status

**(b)** Forest plot showing mean % change in HDL cholesterol for orforglipron administered at doses of daily 3mg, daily 12mg, daily 24mg, daily 36mg, and daily 45mg compared to placebo.

**Supplementary Figure S9:** Effect of Orforglipron vs Placebo on Triglycerides Level


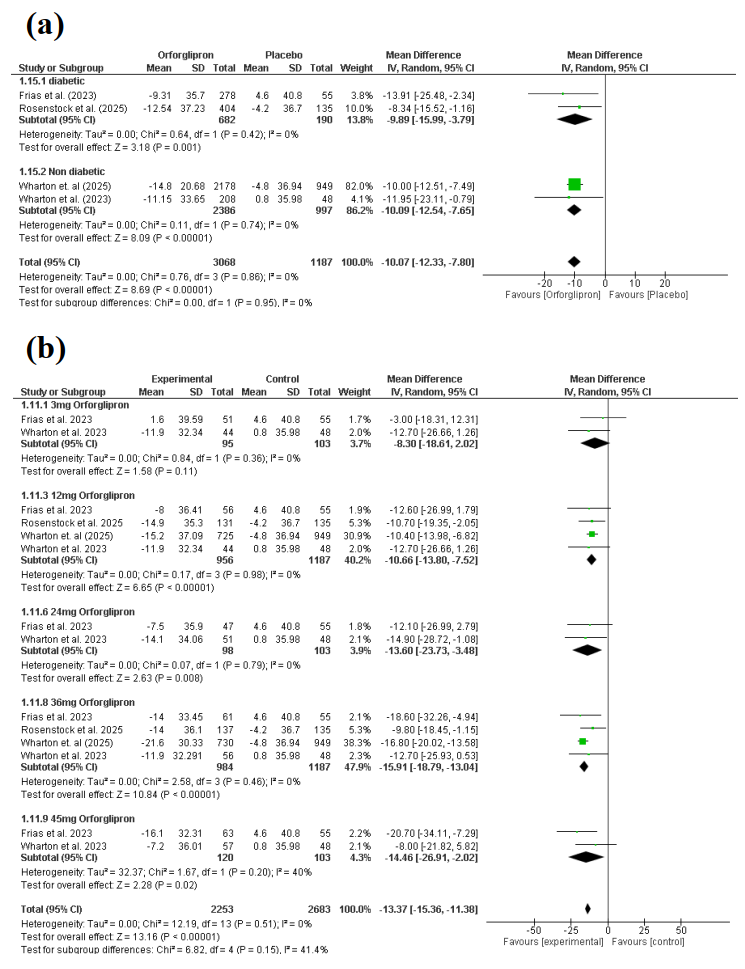


**(a)** Subgroup analysis based on diabetic status

**(b)** Forest plot showing mean % change in Triglycerides for orforglipron administered at doses of daily 3mg, daily 12mg, daily 24mg, daily 36mg, and daily 45mg compared to placebo.

**Supplementary Figure S10:** Effect of Orforglipron vs Placebo


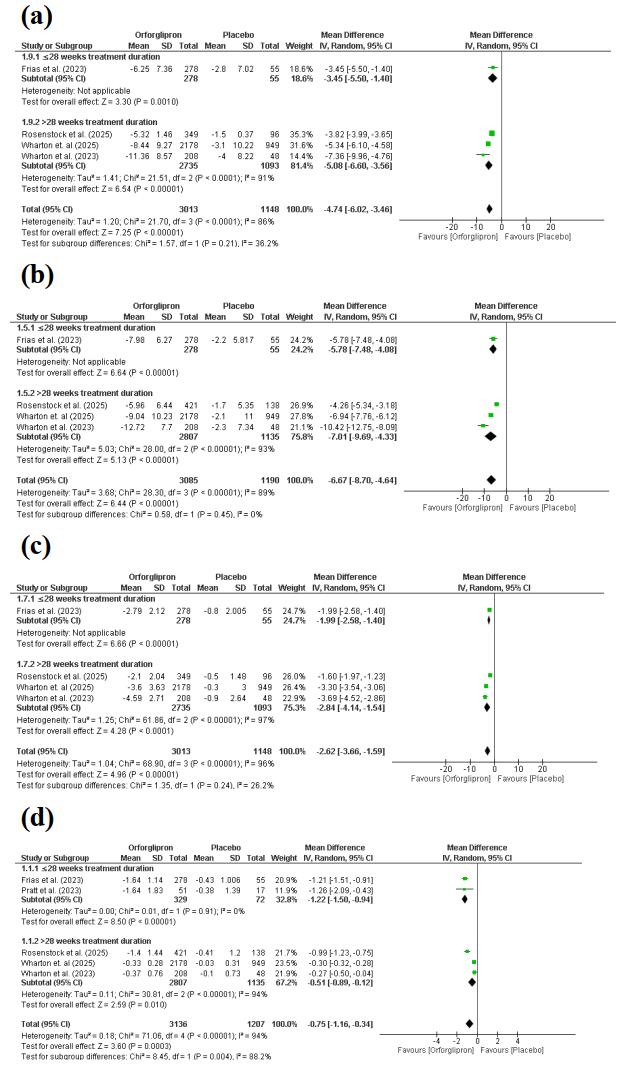


Forest plots showing pooled mean differences between orforglipron and placebo, stratified by treatment duration (≤28 weeks vs. >28 weeks): **(a)** Change in waist circumference (cm), **(b**) percentage change in body weight (%), **(c)** change in BMI (kg/m²), and **(d)** Change in HbA1c (%).
